# Degrees of Freedom of a *K*-User Interference Channel in the Presence of an Instantaneous Relay

**DOI:** 10.3390/e24081078

**Published:** 2022-08-04

**Authors:** Ali H. Abdollahi Bafghi, Mahtab Mirmohseni, Masoumeh Nasiri-Kenari

**Affiliations:** 1 Department of Electrical Engineering, Sharif University of Technology, Tehran P932+FM4, Iran; 2 Institute for Communication Systems (ICS), University of Surrey, Guildford GU2 7XH, UK

**Keywords:** frequency-selective interference channel, K-user interference channel, DoF, instantaneous relay

## Abstract

In this paper, we study the degrees of freedom (DoF) of a frequency-selective *K*-user interference channel in the presence of an instantaneous relay (IR) with multiple receiving and transmitting antennas. We investigate two scenarios based on the IR antennas’ cooperation ability. First, we assume that the IR receiving and transmitting antennas can coordinate with each other and that the transmitted signal of each transmitting antenna can depend on the received signals of all receiving antennas, and we derive lower and upper bounds for the sum DoF of this model. In an interference alignment scheme, we divide receivers into two groups called clean and dirty receivers. We design our scheme such that a part of the messages of clean receivers can be de-multiplexed at the IR. Thus, the IR can use these message streams for an interference cancellation at the clean receivers. Next, we consider an IR, the antennas of which do not have coordination with each other and where the transmitted signal of each transmitting antenna depends only on the received signal of its corresponding receiving antenna. We also derive lower and upper bounds for the sum DoF for this model of IR. We show that the achievable sum DoF decreases considerably compared with the coordinated case. In both of these models, our schemes achieve the maximum *K* sum DoF if the number of transmitting and receiving antennas is more than a finite threshold.

## 1. Introduction

Spectrum sharing in wireless networks seems to be an inevitable solution to increasing bandwidth demands. How to treat interference is one of the main challenges in these scenarios. Interference alignment has proved to be a useful solution that aligns all interference signals into a smaller subspace, allowing the remaining signal space to be used for the transmission of main signals. Thereby, it can achieve the maximum degrees of freedom (DoF) of $\frac{K}{2}$ in a *K*-user interference channel [1]. An interesting question would be to find tools that can improve this maximum value for the DoF. Instantaneous relay (relay-without-delay; IR) is one of these tools [2,3].

For an IR, a transmitted signal in a *t*-th time slot ($X_{\mathrm{IR}}\left(t\right)$) is a function of all received signals ($Y_{\mathrm{IR}}\left(t\right)$) from a first time slot up to a current (*t*-th) time slot, i.e., $Y_{\mathrm{IR}}\left(t\right)=f_{\mathrm{IR}}\left(X_{\mathrm{IR}}\left(1\right),\ldots ,X_{\mathrm{IR}}\left(t\right)\right)$, while for a classic relay, a transmitted signal in a *t*-th time slot does not depend on a received signal in the *t*-th (current) time slot (it was shown in [4] that a classic relay cannot increase the DoF of a *K*-user interference channel), i.e., $Y_{R}\left(t\right)=f_{R}\left(X_{R}\left(1\right),\ldots ,X_{R}\left(t-1\right)\right)$. Though for the current technology, an IR might seem impractical, there have been significant results on an IR, and active reconfigurable intelligent surface (RIS) is a promising technology that makes it possible to realize an IR in the near future [5]. An RIS is a special case of the IR model for which a transmitted signal in the *t*-th time slot ($X_{\mathrm{RIS}}\left(t\right)$) is a function of the received signal ($Y_{\mathrm{RIS}}\left(t\right)$) in the *t*-th time slot only, i.e., $Y_{\mathrm{RIS}}\left(t\right)=f_{\mathrm{RIS}}\left(X_{\mathrm{RIS}}\left(t\right)\right)$.

The capacities of wireless networks in the presence of an IR were studied in [6,7,8,9,10,11,12,13,14,15,16,17,18,19,20,21,22,23,24,25,26,27,28,29,30]. El Gamal et al., in [6], showed that in the presence of an IR, rates higher than an existing cut-set bound for a classic relay can be achieved for a point-to-point channel. In [7], a new upper bound was derived for the capacity of a channel with an IR. The authors in [8] studied a two-user interference channel in the presence of an IR and derived an outer bound for the Gaussian case under strong and very strong interference conditions. They also introduced an achievable scheme based on instantaneous amplify-and-forward relaying. In [9], the authors studied a *K*-user interference channel in the presence of an IR in two scenarios, wherein transmitters and receivers were aware and not aware of the existence of an IR. It was shown that in both cases, an IR can enlarge the rate region and increase user fairness. In [10], the authors studied general networks in the presence of an IR and derived cut-set bounds for two cases of the IR having or not having its own message; they showed that the proposed bounds are tight in some cases. In [11], it was proven that the networks with an IR can be considered a channel with in-block memory. Then, a cut-set bound was characterized that generalizes existing cut-set bounds.

As we stated before, an RIS is a special case of the generic IR model; thus, we will review some related work on the capacities of RIS-assisted networks. In [12], the fundamental capacity limit of RIS-assisted multiple-input multiple-output (MIMO) communications systems was studied by using a joint optimization of a MIMO transmit covariance matrix and RIS phase shifts. In [13], RIS-assisted communication systems were studied wherein a transmitter could control an RIS with a finite-rate link and information-theoretic limits were derived. It was proven that the capacity is achievable if information is jointly encoded in a transmitted signal and RIS phase shifts. In [14], a downlink non-orthogonal multiple-access (NOMA) RIS-assisted communication system was studied wherein multiple users were served by only one base station (BS). The sum rate of the users was maximized by using a joint optimization of a beamforming vector at the BS and the phase shifts of the RIS, wherein a successive interference cancellation decoding rate and RIS scattering element constraints existed. In [15], the usage of an RIS was studied for a rank improvement of MIMO communication channels.

From a DoF perspective, an interference alignment signaling scheme for a MIMO *X*-channel, which outperforms the achievable DoF of previous signaling schemes, was proposed in [16]. It is well known that the DoF of the frequency or time-selective *K*-user interference channel is $\frac{K}{2}$ [1], which is an important result of the interference alignment technique. We remark that the DoF of interference channels is an important problem, which has been studied vastly in the literature; e.g., the DoF of a multi-input multi-output (MIMO) interference channel [17], the DoF region of an interference channel [18,19], and the DoF of an interference channel with a partial network topology [20,21,22,23,24,25]. Interference alignment is an important technique, which has a vital impact on proving DoF achievability theorems for multi-user wireless networks. A survey of the results available on the interference alignment technique was reviewed in [26]. For the DoF of networks in the presence of an IR, the sum DoF of a two-user interference channel assisted by an IR, with *M* antennas for all nodes, was studied in [3], and it was proven that the DoF of $\frac{3M}{2}$ can be achieved. The DoF of an *M* antenna three-user interference channel assisted by an IR was studied in [27], and it was shown that a DoF of 2M is achievable. The DoF of a two-way *K*-user IR-aided interference channel, when the IR is equipped with 2K antennas, was studied in [28]. It was demonstrated that the DoF of *K* can be achieved. The DoF of a two-user interference channel in the presence of an IR, when there is an arbitrary number of IR transmitting and receiving antennas, was studied in [29]. An inner and two outer bounds were obtained. For a *K*-user interference channel assisted by an IR wherein the IR can only instantaneously amplify and forward a received signal in a current channel use, with the same number of antennas at all nodes, an achievable scheme and an outer bound were proposed in [30]. Though the DoF in some special cases wherein K=2 or K(K−1) IRs was derived, a general achievable DoF was not obtained. For a *K*-user interference channel in the presence of active and passive RISs, inner and outer bounds on a DoF region and lower and upper bounds on a sum DoF were derived in [31]. For both active and passive RISs, it was shown that by employing a sufficient number of elements for RIS, a *K* sum DoFs can be achieved. In [32], it was shown that when there is a line-of-sight link between an RIS and transceivers and there is no direct link between the transceivers, the phases of RIS elements can be adjusted such that all interference can be canceled and a maximum *K* DoF can be achieved in a *K*-user interference channel if the number of RIS elements is more than a finite value.

The goal of this paper was to study the sum DoF of a frequency-selective *K*-user interference channel in the presence of an IR. To the best of our knowledge, although the DoF of two- and three-user interference channels and a scenario in which there are K(K−1) IRs have been studied, the sum DoF of a frequency-selective *K*-user interference channel (wherein symbol extensions are in the frequency domain) in the presence of a multi-input multi-output (MIMO) IR has not been characterized. Our contributions are as follows:

- We provide lower and upper bounds for the sum DoF of a *K*-user interference channel in the presence of a MIMO IR with *Q* receiving antennas and *W* transmitting antennas, which can coordinate with each other, i.e., each transmit antenna has access to all receiving antennas. For this purpose, we propose an interference alignment-based coding scheme in which we divide the receivers into two groups called clean and dirty receivers. We design beamforming vectors such that some message symbols corresponding to the clean receivers can be de-multiplexed at the IR. By de-multiplexing, we mean that the IR separates only some of the message symbols using linear operations without removing additive noise. Then, the IR utilizes the de-multiplexed symbols for an interference cancellation at the clean receivers. Our proposed scheme increases the DoF for $W>\frac{K}{2}$ compared to a case without an IR. Moreover, we show that if the number of IR antennas exceeds a finite threshold, the maximum DoF of *K* can be achieved, and we characterize this threshold.
- Moreover, we derive lower and upper bounds for the sum DoF for a special kind of IR for which the IR has the same number of receiving and transmitting antennas and the antennas do not have coordination with each other, i.e., the *i*-th transmitting antenna has access to the *i*-th receiving antenna only. We extend the coding scheme for this case and derive an achievable DoF. Similar to a coordinated IR, we show that by considering a number of IR antennas more than a finite threshold, the maximum DoF of *K* can be achieved. Our derivations show that the achievable DoF decreases considerably compared with the coordinated IR.

This paper is organized as follows. In Section 2, we present the system model. In Section 3 and Section 4, we discuss our main results for the coordinated and non-coordinated IRs, respectively. In Section 5, we present some numerical results to evaluate our proposed schemes. Finally, in Section 6, we conclude the paper.

**Notations:** Bold letters demonstrate matrices. Calligraphic uppercase letters denote sets and vector spaces. R is the set of real numbers. For the set A, |A| indicates the cardinality of A. $V^{T}$ and $V^{H}$ are the transposition and Hermitian of matrix V, respectively. $\mathrm{diag}(a_{1},\ldots ,a_{m})$ denotes a diagonal matrix with the diagonal elements $a_{1},\ldots ,a_{m}$. The function f(ρ) is o(log(ρ)) if

$$\lim_{\rho \to \infty }\frac{\left|f(\rho )\right|}{\log(\rho )}=0.$$

Sequence a(n) goes to infinity with O(g(n)) if

$$0<\lim_{n\to \infty }\frac{\left|a(n)\right|}{\left|g(n)\right|}<\infty .$$

N is the set of natural numbers, and W is the set of non-negative integers.

## 2. System Model and Preliminaries

### 2.1. System Model

**Figure 1:**
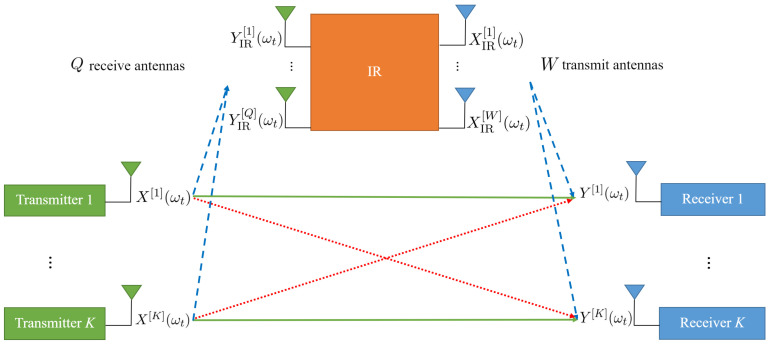
IR-assisted *K*-user interference channel. The IR has *W* transmitting antennas and *Q* receiving antennas. Direct links are shown by solid arrows, cross-links are shown by dotted arrows, and links between the IR and transmitters or receivers are shown by dashed arrows.

We consider a *K*-user interference channel with an IR in which *K* single-antenna transmitters send their messages to *K* single-antenna receivers. In this system, the *i*-th transmitter sends the message $w^{\left[i\right]}\in W^{\left[i\right]}=\left\{1,\ldots ,\left\lfloor 2^{Tr_{i}}\right\rfloor \right\}$ to the *i*-th receiver, where $r_{i}$ is the transmission rate corresponding to the *i*-th transmitter and *T* is the number of channel uses (in this paper, each channel use corresponds to each frequency slot and all transmissions are in the same time cycle). We assume an IR with *Q* receiving antennas and *W* transmitting antennas. Figure 1 shows the system model.

We consider a frequency-selective channel. Due to the instantaneity of the IR, it can process the signals received from all frequency slots in the current time cycle and transmit signals in different frequency slots in the same time cycle, which affects the received signals at the receivers in all frequency slots. The received signal at the *j*-th receiver in the *t*-th frequency slot $\omega_{t}$ is shown by $Y^{\left[j\right]}\left(\omega_{t}\right)$ and is presented as follows (note that in the general case, the IR-transmitted signal is a function of the received signal in the past time cycles in addition to the current time cycle. In the achievability proofs of this paper, the signals of past time cycles are not needed and transmissions in different frequency slots are at the same time cycle. However, for the upper bounds, the general case is considered.):

(1) $$Y^{\left[j\right]}\left(\omega_{t}\right)=\sum_{i=1}^{K}H^{\left[ji\right]}\left(\omega_{t}\right)X^{\left[i\right]}\left(\omega_{t}\right)+\sum_{u=1}^{W}H_{\mathrm{IR}-R}^{\left[ju\right]}\left(\omega_{t}\right)X_{\mathrm{IR}}^{\left[u\right]}\left(\omega_{t}\right)+Z^{\left[j\right]}\left(\omega_{t}\right),$$

where $X^{\left[i\right]}\left(\omega_{t}\right)$ is the signal of the *i*-th transmitter, $H^{\left[ji\right]}\left(\omega_{t}\right)$ is the channel coefficient between the *i*-th transmitter and the *j*-th receiver, $X_{\mathrm{IR}}^{\left[u\right]}\left(\omega_{t}\right)$ is the transmitted signal of the *u*-th IR transmitting antenna, $H_{\mathrm{IR}-R}^{\left[ju\right]}\left(\omega_{t}\right)$ is the channel coefficient between the *u*-th IR transmitting antenna and the *j*-th receiver, and $Z^{\left[j\right]}\left(\omega_{t}\right)$ is additive white Gaussian noise (AWGN) at the *j*-th receiver in the *t*-th frequency slot $\omega_{t}$, where t∈{1,2,…,T}. We assume a perfect self-interference cancellation at the IR; thus, the received signal at the *q*-th IR receiving antenna in the *t*-th frequency slot, which is shown by $Y_{\mathrm{IR}}^{\left[q\right]}\left(\omega_{t}\right)$, is given as follows:

(2) $$Y_{\mathrm{IR}}^{\left[q\right]}\left(\omega_{t}\right)=\sum_{i=1}^{K}H_{T-\mathrm{IR}}^{\left[qi\right]}\left(\omega_{t}\right)X^{\left[i\right]}\left(\omega_{t}\right)+Z_{\mathrm{IR}}^{\left[q\right]}\left(\omega_{t}\right),$$

where $H_{T-\mathrm{IR}}^{\left[qi\right]}\left(\omega_{t}\right)$ is the channel coefficient from the *i*-th transmitter to the *q*-th IR receiving antenna (for an NC-IR, before a transmission begins, all required channel-state information and the transmission strategy are shared between all nodes and all receiving and transmitting antennas of the NC-IR. However, when the transmission begins, the *i*-th transmitting antenna of the NC-IR has access to the *i*-th receiving antenna only and its received signal cannot be exchanged between other transmitting antennas (the same holds for the active RIS [31])), q∈{1,…,Q}, and $Z_{\mathrm{IR}}^{\left[q\right]}\left(\omega_{t}\right)$ are the AWGN at the *q*-th IR receiving antenna in the *t*-th frequency slot. We assume that the perfect channel-state information for all frequency slots is available at all nodes (this ideal assumption is vastly considered in the literature [1,33]. Noisy channel-state information will be an interesting subject of future work.). We consider two types of IR: (1) a MIMO IR, the antennas of which can have a coordination with each other, called MIMO-coordinated IR (C-IR) and (2) an IR with no coordination among its antennas because the *u*-th transmitting antenna has access to only the *u*-th receiving antenna (W=Q). We call this model non-coordinated IR (NC-IR). At each time cycle, for the MIMO C-IR, we have:

(3) $$X_{\mathrm{IR}}^{\left[u\right]}\left(\omega_{t}\right)=f^{\left[u,\omega_{t}\right]}\left(Y_{\mathrm{IR}}^{\left[1\right]}\left(\omega_{1}\right),\ldots ,Y_{\mathrm{IR}}^{\left[1\right]}\left(\omega_{T}\right),\ldots ,Y_{\mathrm{IR}}^{\left[Q\right]}\left(\omega_{1}\right),\ldots ,Y_{\mathrm{IR}}^{\left[Q\right]}\left(\omega_{T}\right)\right),$$

where $f^{\left[u,\omega_{t}\right]}$ indicates the encoding function of the IR for the *u*-th transmitting antenna at the *t*-th frequency slot $\omega_{t}$. For the NC-IR, we have:

(4) $$X_{\mathrm{IR}}^{\left[u\right]}\left(\omega_{t}\right)=f^{\left[u,\omega_{t}\right]}\left(Y_{\mathrm{IR}}^{\left[u\right]}\left(\omega_{1}\right),\ldots ,Y_{\mathrm{IR}}^{\left[u\right]}\left(\omega_{T}\right)\right),u\in \left\{1,\ldots ,Q\right\}.$$

We limit the functions $f^{\left[u,\omega_{t}\right]}$ to be linear. (1) and (2) can be rewritten into the following vector form:

(5) $$Y^{\left[j\right]}=\sum_{i=1}^{K}H^{\left[ji\right]}X^{\left[i\right]}+\sum_{u=1}^{W}H_{\mathrm{IR}-R}^{\left[ju\right]}X_{\mathrm{IR}}^{\left[u\right]}+Z^{\left[j\right]},$$

(6) $$Y_{\mathrm{IR}}^{\left[q\right]}=\sum_{i=1}^{K}H_{T-\mathrm{IR}}^{\left[qi\right]}X^{\left[i\right]}+Z_{\mathrm{IR}}^{\left[q\right]},$$

where $X^{\left[i\right]}$ is a T×1 column vector including the channel inputs $X^{\left[i\right]}\left(\omega_{t}\right)$, i.e.,

$$X^{\left[i\right]}={\left[\begin{array}{cccc} X^{\left[i\right]}\left(\omega_{1}\right) & X^{\left[i\right]}\left(\omega_{2}\right) & \cdots & X^{\left[i\right]}\left(\omega_{T}\right) \end{array}\right]}^{T}.$$

$Y^{\left[i\right]}$, $Y_{\mathrm{IR}}^{\left[q\right]}$, $X_{\mathrm{IR}}^{\left[u\right]}$, $Z^{\left[j\right]}$ and $Z_{\mathrm{IR}}^{\left[q\right]}$ are also defined in the similar way. $H^{\left[ji\right]}$ is a diagonal matrix defined as follows:

$$H^{\left[ji\right]}=\mathrm{diag}\left(H^{\left[ji\right]}\left(\omega_{1}\right),\ldots ,H^{\left[ji\right]}\left(\omega_{T}\right)\right).$$

$H_{\mathrm{IR}-R}^{\left[ju\right]}$ and $H_{T-\mathrm{IR}}^{\left[qi\right]}$ are also defined similarly. Considering functions $f^{\left[u,\omega_{t}\right]}$ to be linear, the operation of the the MIMO C-IR can be represented as follows:

(7) $$X_{\mathrm{IR}}^{\left[u\right]}=\sum_{q=1}^{Q}A^{\left[uq\right]}Y_{\mathrm{IR}}^{\left[q\right]},$$

where $A^{\left[uq\right]}$ are T×T matrices. Moreover, the linear operation of the NC-IR can be represented as follows:

(8) $$X_{\mathrm{IR}}^{\left[u\right]}=A^{\left[u\right]}Y_{\mathrm{IR}}^{\left[u\right]}.$$

Since we assume a frequency-selective *K*-user interference channel, $H^{\left[ji\right]}\left(\omega_{t}\right)$, $H_{\mathrm{IR}-R}^{\left[ju\right]}\left(\omega_{t}\right)$ and $H_{T-\mathrm{IR}}^{\left[qi\right]}\left(\omega_{t}\right)$ are independent random variables for different values of i,j,u,q and $\omega_{t}$, whose cumulative distribution functions (CDFs) are continuous due to the frequency selectivity of the channel. In the case of complex channel coefficients, their real and imaginary parts are independent random variables, whose CDFs are continuous (e.g., complex Gaussian random variable).

**Remark** **1.** *The assumption of frequency selectivity is essential for our coding scheme not only for the realization of independent channel coefficients for each channel use but also because if we assume the channel to be time selective and channel uses are in different time slots, by using (7) and (8), the matrices $A^{\left[uq\right]}$ for the MIMO C-IR and the matrices $A^{\left[u\right]}$ for the NC-IR must be lower triangular matrices due to the definition of the IR (the transmitted signal of an IR for the t-th time slot is a function of the received signals for the time slots $t^{\prime }\in \left\{1,\ldots ,t\right\}$). However, if we assume the channel to be frequency selective and consider our different channel uses in different frequency slots in the same time cycle, the transmitted signals of the IR for each frequency slot can be a function of all received signals for all frequency slots; thus, there would not be any constraint on the matrices $A^{\left[uq\right]}$ and $A^{\left[u\right]}$ and our proposed achievability schemes will be realizable.*

We assume that all transmitters can send a signal with a maximum average power of ρ, i.e., $\frac{1}{T}\sum_{t=1}^{T}{\left|X^{\left[i\right]}\left(\omega_{t}\right)\right|}^{2}$⩽ρ,∀i∈{1,…,K}. We say the rate vector $r=(r_{1},\ldots ,r_{K})$ is achievable if $\lim_{T\to \infty }\mathrm{Pr}\left\{\underset{i}{⋂}\left\{{\hat{w}}^{\left[i\right]}\neq w^{\left[i\right]}\right\}\right\}=0$, where ${\hat{w}}^{\left[i\right]}$ is the estimated message at the *i*-th receiver. In addition, C(ρ) indicates the closure of all the achievable rate vectors $r=(r_{1},\ldots ,r_{K})$.

### 2.2. Preliminaries

In the following section, we introduce some definitions that are used throughout this paper.

**Degrees of freedom (DoF)**: Similar to [1], we define the DoF region D for a *K*-user interference channel as follows:

$$D=\left\{\left(d_{1},\ldots ,d_{K}\right)\in R_{+}^{K}:\forall \left(w_{1},\ldots ,w_{K}\right)\in R_{+}^{K},\right.$$

(9) $$\left.w_{1}d_{1}+\ldots +w_{K}d_{K}\leq \underset{\rho \to \infty }{\lim\sup}\left(\frac{1}{\log(\rho )}\underset{r(\rho )\in C(\rho )}{\sup}\left(w_{1}r_{1}+\ldots +w_{K}r_{K}\right)\right)\right\}.$$

**Span**: Thespan(V) denotes the space spanned by the column vectors of the matrix **V**.

**Dimension**: We define the number of dimensions of thespan(V) as the dimension of **V** and show it by using d(V), which is equal to rank(V).

**Normalized asymptotic dimension**: We will see in our analysis that for a given K,Q and for *W*, the dimensions of the beamforming matrices and the vector spaces will have an order of $O(n^{l}),l,n\in N$. For the matrix **V**, we define the normalized asymptotic dimension ($D_{N}$) as follows:

(10) $$D_{N}\left(V\right)=\lim_{n\to \infty }\frac{d(V)}{n^{l}},$$

where *l* is the maximum integer number that satisfies $\lim_{n\to \infty }\frac{d(V)}{n^{l}}<\infty$.

These definitions are also used for the vector space A; therefore, d(A) indicates the dimension of A, and $D_{N}\left(A\right)$ indicates the normalized asymptotic dimension of A.

## 3. K-User Interference Channel in the Presence of MIMO C-IR

In this section, we present the lower and upper bounds for the sum DoF of the frequency-selective *K*-user interference channel with a MIMO C-IR. First, we introduce the lower bound as follows:

**Theorem** **1.** *For a frequency-selective K-user interference channel with a MIMO C-IR, where max{W,Q}≤K, the following DoF is achievable:*

(11) $$\mathrm{DoF}=\max\left\{\frac{K}{2}+\max\left\{0,K\frac{\frac{W}{K}-\frac{1}{2}}{1+2\left\lceil \frac{W}{Q}\right\rceil }\right\},\min\left\{Q,W\right\}\right\}.$$

*We can see from (11) that when $\frac{W}{K}>\frac{1}{2}$, the DoF always increases over $\frac{K}{2}$, i.e., the DoF increases in the absence of an IR.*

**Proof.**  We will prove the achievability of the first term $\frac{K}{2}+\max\left\{0,K\frac{\frac{W}{K}-\frac{1}{2}}{1+2\left\lceil \frac{W}{Q}\right\rceil }\right\}$ in (11) in the following. The proof of the second term, i.e., min{Q,W}, is provided in Appendix A.

We present this proof in six steps. In Step 1, we divide the transmitters and the receivers into two groups (clean and dirty). In Step 2, some message streams are considered to have the capability of being de-multiplexed at the MIMO C-IR; thus, the MIMO C-IR can use them for an interference cancellation in the clean receivers. After the interference cancellation, the equivalent channel coefficients are derived for other receivers (dirty receivers). In Step 3, we introduce the interference alignment equations such that the assumption of the previous step (the de-multiplexing of some message streams) and the interference alignment for each receiver and MIMO C-IR receiving antenna are satisfied. In Step 4, we present the beamforming design for each symbol stream. In Step 5, we analyze the satisfaction of the interference alignment equations at each receiver and MIMO C-IR receiving antenna. Finally, in Step 6, we derive the achieved DoF, presented in the first term of (11).

**Step 1: Partitioning the Transmitters and Receivers**

We divide the transmitters into two partitions. For the transmitters i∈{1,…,W}, we provide two sets of symbol streams: ${\bar{x}}^{\left[i\right]}$ and ${\tilde{x}}^{\left[i\right]}$ (each element of the vectors ${\bar{x}}^{\left[i\right]}$ and ${\tilde{x}}^{\left[i\right]}$ is the extended symbols). The matrices ${\bar{V}}^{\left[i\right]}$ and ${\tilde{V}}^{\left[i\right]}$ are the beamforming matrices, the columns of which are the beamforming vectors corresponding to the elements of ${\bar{x}}^{\left[i\right]}$ and ${\tilde{x}}^{\left[i\right]}$, respectively. We can write:

(12) $$X^{\left[i\right]}={\bar{V}}^{\left[i\right]}{\bar{x}}^{\left[i\right]}+{\tilde{V}}^{\left[i\right]}{\tilde{x}}^{\left[i\right]},i\in \left\{1,\ldots ,W\right\}.$$

For the transmitters i∈{W+1,…,K}, we only provide one set of extended symbols (${\bar{x}}^{\left[i\right]}$), and ${\bar{V}}^{\left[i\right]}$ is the beamforming matrix for the symbols ${\bar{x}}^{\left[i\right]}$. Thus, we have:

(13) $$X^{\left[i\right]}={\bar{V}}^{\left[i\right]}{\bar{x}}^{\left[i\right]},i\in \left\{W+1,\ldots ,K\right\}.$$

Note that the matrices ${\tilde{V}}^{\left[i\right]}$ and ${\bar{V}}^{\left[i\right]}$ have *T* rows because we have *T* frequency slots. The dimensions of ${\bar{x}}^{\left[i\right]}$ and ${\tilde{x}}^{\left[i\right]}$ and the number of columns of ${\bar{V}}^{\left[i\right]}$ and ${\tilde{V}}^{\left[i\right]}$ are determined in the next steps.

In the following steps, we design the beamforming vectors ${\tilde{V}}^{\left[i\right]}$ and ${\bar{V}}^{\left[i\right]}$ such that the extended symbols ${\tilde{x}}^{\left[i\right]}$ can be de-multiplexed at the MIMO C-IR. By de-multiplexing, we mean that the MIMO C-IR can separate each symbol of message streams ${\tilde{x}}^{\left[i\right]}$ using zero forcing without decoding the symbol. The symbol streams ${\bar{x}}^{\left[i\right]}$ act as interference signals, and their beamforming vectors align into a smaller subspace.

We also divide the receivers into clean and dirty sets. In the next steps, the signal transmitted by the MIMO C-IR is designed such that the interference induced by the symbols ${\tilde{x}}^{\left[i\right]}$ will be removed at the receivers j∈{1,…,W}, called clean receivers, but this interference will remain at the receivers j∈{W+1,…,K}, called dirty receivers. The main reason for choosing these terms (clean and dirty receivers) is that in our scheme, the interference of some symbol streams is canceled at clean receivers by the MIMO C-IR (the MIMO C-IR can de-multiplex these symbols and use them for interference cancellation) and the clean receivers will observe fewer dimensions for the interference; however, all interference remains at the dirty receivers.

**Step 2: Interference Cancellation at Clean Receivers and Equivalent Channel for Dirty Receivers**

We design the beamforming vectors ${\tilde{V}}^{\left[i\right]}$ and ${\bar{V}}^{\left[i\right]}$ such that the interference induced by the symbols ${\tilde{x}}^{\left[i\right]}$ will be removed at the clean receivers. We denote this interference as ${\tilde{I}}^{\left[j\right]}$, which is written as follows:

(14) $${\tilde{I}}^{\left[j\right]}=\sum_{i\in \{1,\ldots ,W\},i\neq j}H^{\left[ji\right]}{\tilde{V}}^{\left[i\right]}{\tilde{x}}^{\left[i\right]},j\in \left\{1,\ldots ,W\right\},$$

The MIMO C-IR can de-multiplex the streams corresponding to ${\tilde{x}}^{\left[i\right]}$ (this will be shown in Steps 3–5), which is only contaminated by an additive noise, i.e., it will separate them into the form of ${\hat{\tilde{x}}}^{\left[i\right]}={\tilde{x}}^{\left[i\right]}+{\tilde{z}}^{\left[i\right]}$. Thus, for the interference cancellation, the MIMO C-IR designs its transmitted signal such that:

$$\sum_{u\in \{1,\ldots ,W\}}H_{\mathrm{IR}-R}^{\left[ju\right]}X_{\mathrm{IR}}^{\left[u\right]}=$$

(15) $$-\sum_{i\in \{1,\ldots ,W\},i\neq j}H^{\left[ji\right]}{\tilde{V}}^{\left[i\right]}{\hat{\tilde{x}}}^{\left[i\right]}=-\sum_{i\in \{1,\ldots ,W\},i\neq j}H^{\left[ji\right]}{\tilde{V}}^{\left[i\right]}\left({\tilde{x}}^{\left[i\right]}+{\tilde{z}}^{\left[i\right]}\right)=-{\tilde{I}}^{\left[j\right]}+{\tilde{Z}}^{\left[j\right]},$$

where

$${\tilde{Z}}^{\left[j\right]}=-\sum_{i\in \{1,\ldots ,W\},i\neq j}H^{\left[ji\right]}{\tilde{V}}^{\left[i\right]}{\tilde{z}}^{\left[i\right]}.$$

The vector Equation (15) generates a linear set of equations, an equation for each element of $X_{\mathrm{IR}}^{\left[u\right]}$, which can be written for the *t*-th element as:

(16) $$\sum_{u\in \{1,\ldots ,W\}}H_{\mathrm{IR}-R}^{\left[ju\right]}\left(\omega_{t}\right)X_{\mathrm{IR}}^{\left[u\right]}\left(\omega_{t}\right)=-{\tilde{I}}^{\left[j\right]}\left(\omega_{t}\right)+{\tilde{Z}}^{\left[j\right]}\left(\omega_{t}\right),\;\forall j\in \left\{1,\ldots ,W\right\},\;\forall t\in \left\{1,\ldots ,T\right\},$$

which is a linear set of equations with *W* variables for each $\omega_{t}$. This set of equations is almost surely solvable since the coefficients of the linear equations are drawn independently and their CDFs are continuous; thus, the determinant of the matrix of linear equations will be a non-zero polynomial in terms of independent random variables and by using ([34], Lemma 1), it will be a non-zero with a probability equal to 1. Applying (16), the interference cancellation will be conducted. Thus, for each $\omega_{t}$, we will have:

(17) $$X_{\mathrm{IR}}^{\left[u\right]}\left(\omega_{t}\right)=\sum_{j\in \{1,\ldots ,W\}}H_{\mathrm{inv}}^{\left[ju\right]}\left(\omega_{t}\right)\left(-{\tilde{I}}^{\left[j\right]}\left(\omega_{t}\right)\left(\omega_{t}\right)+{\tilde{Z}}^{\left[j\right]}\left(\omega_{t}\right)\right),$$

where $H_{\mathrm{inv}}^{\left[ju\right]}\left(\omega_{t}\right)$, the factor of $-{\tilde{I}}^{\left[j\right]}\left(\omega_{t}\right)+{\tilde{Z}}^{\left[j\right]}\left(\omega_{t}\right)$ in (17), is a function of $H_{\mathrm{IR}-R}^{\left[j^{\prime }u^{\prime }\right]}\left(\omega_{t}\right),u^{\prime },j^{\prime }\in \left\{1,\ldots ,W\right\}$ obtained by solving Equation (16). We can write Equation (17) in the vector form as follows:

(18) $$\begin{array}{cc} X_{\mathrm{IR}}^{\left[u\right]} & =\sum_{j\in \{1,\ldots ,W\}}H_{\mathrm{inv}}^{\left[ju\right]}\left(-{\tilde{I}}^{\left[j\right]}+{\tilde{Z}}^{\left[j\right]}\right) \end{array}$$

(19) $$\begin{array}{cc} \; & =\sum_{j\in \{1,\ldots ,W\}}\sum_{i\in \{1,\ldots ,W\},i\neq j}-H_{\mathrm{inv}}^{\left[ju\right]}H^{\left[ji\right]}{\tilde{V}}^{\left[i\right]}{\tilde{x}}^{\left[i\right]}+\sum_{j\in \{1,\ldots ,W\}}H_{\mathrm{inv}}^{\left[ju\right]}{\tilde{Z}}^{\left[j\right]}, \end{array}$$

where $H_{\mathrm{inv}}^{\left[ju\right]}$ is a diagonal matrix as follows:

$$H_{\mathrm{inv}}^{\left[ju\right]}=\mathrm{diag}\left(H_{\mathrm{inv}}^{\left[ju\right]}\left(\omega_{1}\right),\ldots ,H_{\mathrm{inv}}^{\left[ju\right]}\left(\omega_{T}\right)\right).$$

We highlight two properties of $H_{\mathrm{inv}}^{\left[ju\right]}$:

- Similar to $H^{\left[ji\right]}$, diagonal elements $H_{\mathrm{inv}}^{\left[ju\right]}\left(\omega_{t}\right)$ are independent random variables for different t∈{1,…,T} because the channel coefficients are independent random variables for each t∈{1,…,T}.
- Each diagonal element $H_{\mathrm{inv}}^{\left[ju\right]}\left(\omega_{t}\right)$ is a fractional polynomial constructed by the matrices $H_{\mathrm{IR}-R}^{\left[j^{\prime }u^{\prime }\right]}\left(\omega_{t}\right),j^{\prime },u^{\prime }\in \left\{1,\ldots ,W\right\}$. A fractional polynomial is the ratio of the polynomial $P_{1}\left(\cdot \right)$ to the non-zero polynomial $P_{2}\left(\cdot \right)$.

Although we cancel the interference ${\tilde{I}}^{\left[j\right]}$ at the clean receivers, this interference remains at the dirty receivers with new equivalent channel coefficients. Now, we derive the new channel coefficients for ${\tilde{V}}^{\left[i\right]}{\tilde{x}}^{\left[i\right]},\forall i\in \left\{1,\ldots ,W\right\}$ at the dirty receivers j∈{W+1,…,K}. By combining (5), (12), and (13), we have:

(20) $$\begin{array}{cc} Y^{\left[j\right]} & =\sum_{i\in \{1,\ldots ,K\}}H^{\left[ji\right]}{\bar{V}}^{\left[i\right]}{\bar{x}}^{\left[i\right]}+\sum_{i\in \{1,\ldots ,W\}}H^{\left[ji\right]}{\tilde{V}}^{\left[i\right]}{\tilde{x}}^{\left[i\right]}+\sum_{u\in \{1,\ldots ,W\}}H_{\mathrm{IR}-R}^{\left[ju\right]}X_{\mathrm{IR}}^{\left[u\right]}+Z^{\left[j\right]} \end{array}$$

(21) $$\begin{array}{cc} \; & =\sum_{i\in \{1,\ldots ,K\}}H^{\left[ji\right]}{\bar{V}}^{\left[i\right]}{\bar{x}}^{\left[i\right]}+\sum_{i\in \{1,\ldots ,W\}}H^{\left[ji\right]}{\tilde{V}}^{\left[i\right]}{\tilde{x}}^{\left[i\right]}+\sum_{u,d,i\in \{1,\ldots ,W\},i\neq d}H_{\mathrm{IR}-R}^{\left[ju\right]}H_{\mathrm{inv}}^{\left[du\right]}H^{\left[di\right]}{\tilde{V}}^{\left[i\right]}{\tilde{x}}^{\left[i\right]}+{\tilde{\tilde{Z}}}^{\left[j\right]}, \end{array}$$

where (21) follows from (19) and:

$${\tilde{\tilde{Z}}}^{\left[j\right]}=\sum_{u,d}H_{\mathrm{IR}-R}^{\left[ju\right]}H_{\mathrm{inv}}^{\left[du\right]}{\tilde{Z}}^{\left[d\right]}+Z^{\left[j\right]}.$$

(21) can be rewritten as:

(22) $$Y^{\left[j\right]}=\sum_{i\in \{1,\ldots ,K\}}H^{\left[ji\right]}{\bar{V}}^{\left[i\right]}{\bar{x}}^{\left[i\right]}+\sum_{i\in \{1,\ldots ,W\}}{\tilde{H}}^{\left[ji\right]}{\tilde{V}}^{\left[i\right]}{\tilde{x}}^{\left[i\right]}+{\tilde{\tilde{Z}}}^{\left[j\right]},$$

(23) $${\tilde{H}}^{\left[ji\right]}=H^{\left[ji\right]}+\sum_{u,d\in \{1,\ldots ,W\},d\neq i}H_{\mathrm{IR}-R}^{\left[ju\right]}H_{\mathrm{inv}}^{\left[du\right]}H^{\left[di\right]},i\in \left\{1,\ldots ,W\right\},$$

where ${\tilde{H}}^{\left[ji\right]}$ is the equivalent channel coefficient matrix from the transmitter i∈{1,…,W} to the receiver j∈{W+1,…,K} (dirty receivers) for ${\tilde{V}}^{\left[i\right]}{\tilde{x}}^{\left[i\right]}$. By using (23), we can see that ${\tilde{H}}^{\left[ji\right]}$ has the following properties:

- ${\tilde{H}}^{\left[ji\right]}$ is a diagonal matrix.
- ${\tilde{H}}^{\left[ji\right]}=H^{\left[ji\right]},\forall j\in \left\{1,\ldots ,W\right\}$.
- For j∈{W+1,…,K}, its *t*-th diagonal element has the following form:
  $${\tilde{H}}^{\left[ji\right]}\left(\omega_{t}\right)=$$
  $$\sum_{u,i^{\prime },j^{\prime }\in \left\{1,\ldots W\right\},i^{\prime }\neq j^{\prime }}H_{\mathrm{IR}-R}^{\left[ju\right]}\left(\omega_{t}\right)H^{\left[j^{\prime }i^{\prime }\right]}\left(\omega_{t}\right)P^{\left[ui^{\prime }j^{\prime }\right]}\left(\left\{H_{\mathrm{IR}-R}^{\left[me\right]}\left(\omega_{t}\right):m,e\in \left\{1,\ldots ,W\right\}\right\}\right)+H^{\left[ji\right]}\left(\omega_{t}\right),$$
  where $P^{\left[ui^{\prime }j^{\prime }\right]}\left(S\right)$ indicates a fractional polynomial constructed from the variables s∈S.

**Step 3: Interference Alignment**

In this step, we determine the interference alignment equations in the clean and dirty receivers and MIMO C-IR receiving antennas. In our interference alignment scheme, we align the subspace of the interference of each user into a bigger subspace with an equal normalized asymptotic dimension. Note that for the matrices **V** and $V^{\prime }$, we can have the following relations simultaneously: $d\left(V\right)>d\left(V^{\prime }\right)$, $D_{N}\left(V\right)=D_{N}\left(V^{\prime }\right)$, e.g., $d\left(V\right)={\left(n+1\right)}^{l}>d\left(V^{\prime }\right)=n^{l}$, $D_{N}\left(V\right)=D_{N}\left(V^{\prime }\right)=1$. We begin with clean receivers.

*(1) Interference alignment at clean receivers:* Consider the clean receiver j∈{1,…,W}; for each i∈{1,…,K},i≠j, we must have:

(24) $$\mathrm{span}\left(H^{\left[ji\right]}{\bar{V}}^{\left[i\right]}\right)\subseteq {\bar{A}}_{j},$$

where ${\bar{A}}_{j}$ is considered a subspace that encompass all interference at the *j*-th receiver induced by ${\bar{x}}^{\left[i\right]},i\in \left\{1,\ldots ,K\right\},i\neq j$, for which we have:

(25) $$\max_{i\in \{1,\ldots ,K\},i\neq j}D_{N}\left(\mathrm{span}\left(H^{\left[ji\right]}{\bar{V}}^{\left[i\right]}\right)\right)=D_{N}\left({\bar{A}}_{j}\right),$$

which implies that the normalized asymptotic dimension of ${\bar{A}}_{j}$ is equal to the maximum asymptotic dimension of $\mathrm{span}\left(H^{\left[ji\right]}{\bar{V}}^{\left[i\right]}\right)$ for ∀i≠j. Moreover, we define the message subspaces as:

$${\bar{C}}_{j}=\mathrm{span}\left(H^{\left[jj\right]}{\bar{V}}^{\left[j\right]}\right),$$

$${\tilde{C}}_{j}=\mathrm{span}\left({\tilde{H}}^{\left[jj\right]}{\tilde{V}}^{\left[j\right]}\right).$$

and we require ${\bar{C}}_{j}$, ${\tilde{C}}_{j}$ and ${\bar{A}}_{j}$ to be full-rank and linearly independent; thus, we can ensure the decodability of the message streams ${\tilde{x}}^{\left[j\right]}$ and ${\bar{x}}^{\left[j\right]}$ by using zero forcing at the *j*-th receiver.

*(2) Interference alignment at dirty receivers:* Consider the dirty receiver j∈{W+1,…,K}. Here, we have two interference subspaces at each receiver *j*; the interference induced by ${\bar{x}}^{\left[i\right]}$ aligns in subspace ${\bar{A}}_{j}$, while the interference induced by ${\tilde{x}}^{\left[i\right]}$ aligns in subspace ${\tilde{A}}_{j}$. For each i∈{1,…,K},i≠j, we must have:

(26) $$\mathrm{span}\left(H^{\left[ji\right]}{\bar{V}}^{\left[i\right]}\right)\subseteq {\bar{A}}_{j},$$

where ${\bar{A}}_{j}$ is considered a subspace for which we have:

(27) $$\max_{i\in \{1,\ldots ,K\},i\neq j}D_{N}\left(\mathrm{span}\left(H^{\left[ji\right]}{\bar{V}}^{\left[i\right]}\right)\right)=D_{N}\left({\bar{A}}_{j}\right),$$

and for every i∈{1,…,W}, we must have:

(28) $$\mathrm{span}\left({\tilde{H}}^{\left[ji\right]}{\tilde{V}}^{\left[i\right]}\right)\subseteq {\tilde{A}}_{j},$$

where ${\tilde{A}}_{j}$ is considered a subspace for which we have:

(29) $$\max_{i\in \{1,\ldots ,W\}}D_{N}\left(\mathrm{span}\left({\tilde{H}}^{\left[ji\right]}{\tilde{V}}^{\left[i\right]}\right)\right)=D_{N}\left({\tilde{A}}_{j}\right).$$

Moreover, we define the message subspace as:

$${\bar{C}}_{j}=\mathrm{span}\left(H^{\left[jj\right]}{\bar{V}}^{\left[j\right]}\right),$$

and we want ${\bar{C}}_{j}$, ${\tilde{A}}_{j}$ and ${\bar{A}}_{j}$ to be full-rank and linearly independent; hence, we can ensure the decodability of the message stream ${\bar{x}}^{\left[j\right]}$ by using zero forcing in the *j*-th receiver.

*(3) Interference alignment at the MIMO C-IR q-th receiving antenna:* We assume that W=QZ+P,0≤P<Q; we divide the transmitters i∈{1,…,W}, into *Q* distinct sets, and the first *P* sets include Z+1 transmitters and the other Q−P sets include *Z* transmitters. We name these sets $B_{q},q\in \left\{1,\ldots ,Q\right\}$. We designed our interference alignment scheme such that the symbol streams ${\tilde{x}}^{\left[i\right]},i\in B_{q}$ can be de-multiplexed at the *q*-th receiving antenna of the MIMO C-IR. To this end, all the interference induced by the symbol streams ${\bar{x}}^{\left[i\right]},i\in \left\{1,\ldots ,K\right\}$ must align into a limited subspace at each receiving antenna of the MIMO C-IR. Thus, at each receiving antenna q∈{1,…,Q}, and for each i∈{1,…,K}, we must have:

(30) $$\mathrm{span}\left(H_{T-\mathrm{IR}}^{\left[qi\right]}{\bar{V}}^{\left[i\right]}\right)\subseteq {\bar{A}}_{r_{q}},$$

where ${\bar{A}}_{r_{q}}$ is considered a subspace for which we have:

(31) $$\max_{i\in \{1,\ldots ,K\}}D_{N}\left(\mathrm{span}\left(H_{T-\mathrm{IR}}^{\left[qi\right]}{\bar{V}}^{\left[i\right]}\right)\right)=D_{N}\left({\bar{A}}_{r_{q}}\right).$$

In addition, at the *q*-th receiving antenna of the MIMO C-IR, the interference induced by the symbol streams ${\tilde{x}}^{\left[i\right]},i\in \left\{1,\ldots ,W\right\},i\notin B_{q}$ must align into a subspace named ${\tilde{A}}_{r_{q}}$. Hence, for each $i\in \left\{1,\ldots ,W\right\},i\notin B_{q}$, we must have:

(32) $$\mathrm{span}\left(H_{T-\mathrm{IR}}^{\left[qi\right]}{\tilde{V}}^{\left[i\right]}\right)\subseteq {\tilde{A}}_{r_{q}},$$

where ${\tilde{A}}_{r_{q}}$ is considered a subspace for which we have:

(33) $$\max_{i\in \left\{1,\ldots ,W\right\},i\notin B_{q}}D_{N}\left(\mathrm{span}\left(H_{T-\mathrm{IR}}^{\left[qi\right]}{\tilde{V}}^{\left[i\right]}\right)\right)=D_{N}\left({\tilde{A}}_{r_{q}}\right).$$

Furthermore, we define ${\tilde{C}}_{i,r_{q}},i\in B_{q}$ as the message subspaces, which can be de-multiplexed at the *q*-th MIMO C-IR receiving antenna as follows:

$${\tilde{C}}_{i,r_{q}}=\mathrm{span}\left(H_{T-\mathrm{IR}}^{\left[qi\right]}{\tilde{V}}^{\left[i\right]}\right),i\in B_{q}.$$

**Figure 2:**
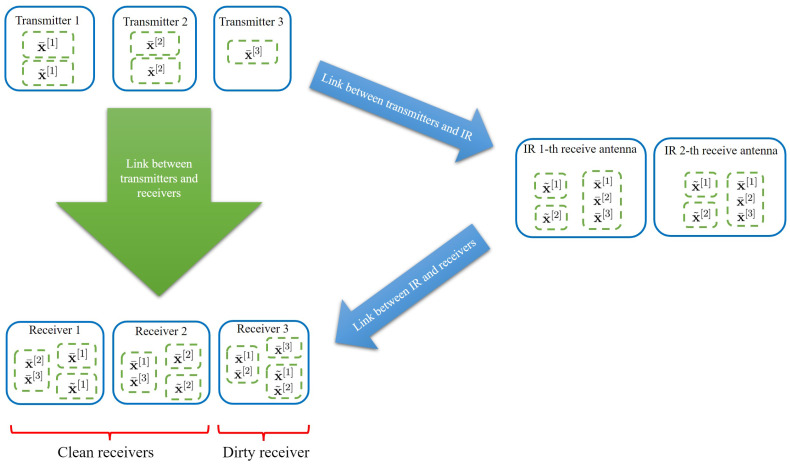
Interference alignment scheme for 3-user interference channel in the presence of MIMO C-IR with 2 receiving antennas. Subspaces corresponding to symbol streams in common dashed boxes align into a joint subspace at each node. We can see that the interference of the message streams ${\tilde{x}}^{\left[1\right]}$ and ${\tilde{x}}^{\left[2\right]}$ is canceled at clean receivers.

We want ${\tilde{C}}_{i,r_{q}},\forall i\in B_{q}$, ${\bar{A}}_{r_{q}}$ and ${\tilde{A}}_{r_{q}}$ to be full-rank and linearly independent; thus, we can make sure that the message streams ${\tilde{x}}^{\left[i\right]},i\in B_{q}$ can be de-multiplexed at the *q*-th MIMO C-IR receiving antenna by using zero forcing. Note that the *q*-th receiving antenna of the MIMO C-IR de-multiplexes the message streams ${\tilde{x}}^{\left[i\right]},i\in B_{q}$ without having the coordination with other receiving antennas. After each antenna de-multiplexes its own message streams ${\tilde{x}}^{\left[i\right]},i\in B_{q}$, all of these message streams are passed to the MIMO C-IR transmitting antennas so the transmitting antennas can have coordination with each other for an interference cancellation at the clean receivers (as in Equation (19)). A simple illustration of the interference alignment scheme is shown in Figure 2 for K=3 and W=2. In Steps 4 and 5, we prove the existence of such beamforming vectors, messages, and interference subspaces, which satisfies the previous interference alignment Equations (24)–(33) for the clean and dirty receivers and the MIMO C-IR. In Step 6, we analyze the achieved DoF by using these beamforming vector designs.

**Step 4: Beamforming Matrix Design**

In this step, we design beamforming matrices such that the alignment Equations (24)–(33) are satisfied and all users’ message streams are decodable.

*(1) Beamforming matrix design for i∈{1,…,W}*: To introduce the beamforming matrix design, we must define some new notations. First, we define set F(A,B) as the set of all functions g(x):A→B, i.e.,

(34) F(A,B)={g(x)|g(x):A→B}.

It is obvious that $\left|F\left(A,B\right)\right|={\left|A\right|}^{\left|B\right|}$. Moreover, we define matrix $M(g\left(x\right),N^{\left[x\right]},A)$ as follows:

(35) $$M\left(g\left(x\right),N^{\left[x\right]},A\right)=\prod_{x\in A}{\left(N^{\left[x\right]}\right)}^{g(x)}.$$

Then, consider the vector $w={\left[\begin{array}{cccc} 1 & 1 & \cdots & 1 \end{array}\right]}^{H}$. We design the beamforming matrices ${\bar{V}}^{\left[i\right]}$ and ${\tilde{V}}^{\left[i\right]}$ as the following:

$${\bar{V}}^{\left[i\right]}=\left\{\left[M(g_{1}\left(i,j\right),H^{\left[ji\right]},{\bar{S}}_{1})\right]\left[M(g_{2}\left(i,q\right),H_{T-\mathrm{IR}}^{\left[qi\right]},{\bar{S}}_{2})\right]w:\right.$$

(36) $$\left.g_{1}\in F\left({\bar{S}}_{1},\left\{1,\ldots ,n\right\}\right),g_{2}\in F\left({\bar{S}}_{2},\left\{1,\ldots ,sn\right\}\right)\right\},$$

where

(37) $${\bar{S}}_{1}=\left\{\left(i,j\right)\left|i,j\in \{1,\ldots ,K\},i\neq j\right.\right\},$$

(38) $${\bar{S}}_{2}=\left\{\left(i,q\right)\left|i\in \{1,\ldots ,K\},q\in \{1,\ldots ,Q\}\right.\right\},$$

where n∈N is an auxiliary variable that can go to infinity, and *s* is a parameter for controlling the dimension of ${\bar{V}}^{\left[i\right]}$, i.e., $d({\bar{V}}^{\left[i\right]})$. This notation means that the right-hand side of (36) is the set of column vectors, which forms the beamforming matrix ${\bar{V}}^{\left[i\right]}$. For ${\tilde{V}}^{\left[i\right]}$, we have:

$${\tilde{V}}^{\left[i\right]}=\left\{\left[M(g_{1}\left(i,j\right),{\tilde{H}}^{\left[ji\right]},{\bar{S}}_{1})\right]\left[M(g_{2}\left(i,q\right),H_{T-\mathrm{IR}}^{\left[qi\right]},{\tilde{S}}_{2})\right]\left[M(g_{3}\left(i,q\right),T^{\left[qi\right]},{\tilde{S}}_{3})\right]w:\right.$$

(39) $$g_{1}\in F\left({\bar{S}}_{1},\left\{1,\ldots ,n\right\}\right),g_{2}\in F\left({\tilde{S}}_{2},\left\{1,\ldots ,sn\right\}\right)\left.,g_{3}\in F\left({\tilde{S}}_{3},\left\{1,\ldots ,\upsilon n\right\}\right)\right\},$$

where ${\bar{S}}_{1}$ is given in (37), and we have:

(40) $${\tilde{S}}_{2}=\left\{\left(i,q\right)\left|i\in \left\{1,\ldots ,K\right\},i\notin B_{q},q\in \left\{1,\ldots ,Q\right\}\right.\right\},$$

(41) $${\tilde{S}}_{3}=\left\{\left(i,q\right)\left|i\in B_{q},q\in \left\{1,\ldots ,Q\right\}\right.\right\},$$

$T^{\left[qi\right]}$s are T×T diagonal random matrices for each *i* and *q*, where each of the diagonal elements for each matrix is drawn independently and its CDF is continuous.

*(2) Beamforming matrix design for i∈{W+1,…,K}*: We consider the beamforming matrix ${\bar{V}}^{\left[i\right]}$ as the following:

$${\bar{V}}^{\left[i\right]}=\left\{\left[M(g_{1}\left(i,j\right),H^{\left[ji\right]},{\bar{S}}_{1})\right]\left[M(g_{2}\left(i,q\right),H_{T-\mathrm{IR}}^{\left[qi\right]},{\bar{S}}_{2})\right]w:\right.$$

(42) $$\left.g_{1}\in F\left({\bar{S}}_{1},\left\{1,\ldots ,n\right\}\right),g_{2}\in F\left({\bar{S}}_{2},\left\{1,\ldots ,tn\right\}\right)\right\},$$

where ${\bar{S}}_{1}$ and ${\bar{S}}_{2}$ are given by using (37) and (38), respectively. *t* is a parameter for controlling the dimension of ${\bar{V}}^{\left[i\right]}$, i.e., $d({\bar{V}}^{\left[i\right]})$.

We note that each value of parameters s,υ and *t* can be approximated by using rational numbers with arbitrarily small errors, and by choosing a sufficiently large *n*, parameters sn,υn and tn will be integers and our proposed scheme will be realizable.

**Step 5: Validity of Interference Alignment Conditions and Decodability of Message Symbols**

Now, we analyze the spaces of messages and interference.

*(1) Validity of interference alignment conditions at the clean receivers j∈{1,…,W}:* For the clean receivers j∈{1,…,W}, we have the following lemma:

**Lemma** **1.** *For the clean receivers j∈{1,…,W}, consider ${\bar{C}}_{j}$ as the message subspace corresponding to the symbol stream ${\bar{x}}^{\left[j\right]}$, consider ${\tilde{C}}_{j}$ as the message subspace corresponding to the symbol stream ${\tilde{x}}^{\left[j\right]}$, and consider ${\bar{A}}_{j}$ as the interference subspace induced by the symbol stream ${\bar{x}}^{\left[j^{\prime }\right]},j^{\prime }\neq j$. Then, ${\bar{C}}_{j},$ ${\tilde{C}}_{j}$ and ${\bar{A}}_{j}$ are full-rank and linearly independent, i.e., all base vectors of these subspaces are linearly independent. Thus, the message streams ${\bar{x}}^{\left[j\right]}$ and ${\tilde{x}}^{\left[j\right]}$ are decodable by using zero forcing. In addition, we have:*

(43) $$D_{N}\left({\bar{C}}_{j}\right)=\Gamma ,$$

(44) $$D_{N}\left({\tilde{C}}_{j}\right)=\chi ,$$

(45) $$D_{N}\left({\bar{A}}_{j}\right)=\max\left\{\Gamma ,\zeta \right\},$$

*where*

$$\Gamma =s^{QK},\;\chi =s^{QK-W}\upsilon^{W},\;\zeta =t^{QK}.$$

**Proof.**  The proof is provided in Appendix B.  □

*(2) Validity of interference alignment conditions at the dirty receivers j∈{W+1,…,K}:* For the dirty receivers j∈{W+1,…,K}, we have the following lemma:

**Lemma** **2.** *For the dirty receivers j∈{W+1,…,K}, consider ${\bar{C}}_{j}$ the message subspace corresponding to the symbol stream ${\bar{x}}^{\left[j\right]}$, consider ${\tilde{A}}_{j}$ as the interference subspace corresponding to the symbol stream ${\tilde{x}}^{\left[j^{\prime }\right]},j^{\prime }\neq j$, and consider ${\bar{A}}_{j}$ as the interference subspace induced by the symbol streams ${\bar{x}}^{\left[j^{\prime }\right]},j^{\prime }\neq j$. Then, ${\bar{C}}_{j},{\tilde{A}}_{j}$ and ${\bar{A}}_{j}$ are full-rank and linearly independent, i.e., all base vectors of these subspaces are linearly independent. Thus, the message stream ${\bar{x}}^{\left[j\right]}$ is decodable by using zero forcing. In addition, we have:*

(46) $$D_{N}\left({\bar{C}}_{j}\right)=\zeta ,$$

(47) $$D_{N}\left({\bar{A}}_{j}\right)=\max\left\{\Gamma ,\zeta \right\},$$

(48) $$D_{N}\left({\tilde{A}}_{j}\right)=\chi .$$

**Proof.**  The proof is provided in Appendix C.  □

*(3) Validity of interference alignment conditions at the MIMO C-IR q-th receiving antenna q∈{1,…,Q}:* For the *q*-th receiving antenna of the MIMO C-IR q∈{1,…,Q}, we have the following lemma:

**Lemma** **3.** *For the q-th receiving antenna of the MIMO C-IR q∈{1,…,Q}, consider ${\tilde{C}}_{i,r_{q}}$ the message subspace corresponding to the symbol streams ${\tilde{x}}^{\left[i\right]},i\in B_{q}$, consider ${\tilde{A}}_{r_{q}}$ the interference subspace corresponding to the symbol streams ${\tilde{x}}^{\left[j\right]},j\neq B_{q}$, and consider ${\bar{A}}_{r_{q}}$ the interference subspace induced by the symbol streams ${\bar{x}}^{\left[j\right]},\forall j$. Then, ${\tilde{C}}_{i,r_{q}},i\in B_{q}$, ${\bar{A}}_{r_{q}}$, and ${\tilde{A}}_{r_{q}}$ are full-rank and linearly independent, i.e., all base vectors of these subspaces are linearly independent. Thus, the message stream ${\tilde{x}}^{\left[i\right]},i\in B_{q}$ can be de-multiplexed by using zero forcing. In addition, we have:*

(49) $$D_{N}\left({\tilde{C}}_{i,r_{q}}\right)=\chi ,$$

(50) $$\sum_{i\in B_{q}}D_{N}\left({\tilde{C}}_{i,r_{q}}\right)=\left|B_{q}\right|\chi ,$$

(51) $$D_{N}\left({\bar{A}}_{r_{q}}\right)=\max\left\{\Gamma ,\zeta \right\},$$

(52) $$D_{N}\left({\tilde{A}}_{r_{q}}\right)=\chi .$$

**Proof.**  The proof is provided in Appendix D.  □

Now, we can calculate the dimension of the whole signal space at each receiver. We define $d_{t,j}$ as the total dimension at the *j*-th receiver and $d_{t,r_{q}}$ as the total dimension at the *q*-th receiving antenna of the MIMO C-IR; thus, we have:

(53) $$d_{t,j}=d\left({\bar{C}}_{j}\right)+d\left({\tilde{C}}_{j}\right)+d\left({\bar{A}}_{j}\right),\forall j\in \left\{1,\ldots ,W\right\},$$

(54) $$d_{t,j}=d\left({\bar{C}}_{j}\right)+d\left({\bar{A}}_{j}\right)+d\left({\tilde{A}}_{j}\right),\forall j\in \left\{W+1,\ldots ,K\right\},$$

(55) $$d_{t,r_{q}}=\sum_{i\in B_{q}}d({\tilde{C}}_{i,r_{q}})+d\left({\bar{A}}_{r_{q}}\right)+d\left({\tilde{A}}_{r_{q}}\right),\forall q\in \left\{1,\ldots ,Q\right\},$$

where the dimension of the message and the interference subspaces are derived in (A8)–(A10), (A20)–(A22), and (A26)–(A28) in Appendix B,  Appendix C and Appendix D. Similarly, define $D_{N,t,j}$ as the total normalized asymptotic dimension at the *j*-th receiver and $D_{N,t,r_{q}}$ as the total normalized asymptotic dimension at the *q*-th receiving antenna of the MIMO C-IR; thus, from (43)–(52), we have:

(56) $$D_{N,t,j}=D_{N}\left({\bar{C}}_{j}\right)+D_{N}\left({\tilde{C}}_{j}\right)+D_{N}\left({\bar{A}}_{j}\right)=\Gamma +\chi +\max\left\{\Gamma ,\zeta \right\},\forall j\in \left\{1,\ldots ,W\right\},$$

(57) $$D_{N,t,j}=D_{N}\left({\bar{C}}_{j}\right)+D_{N}\left({\bar{A}}_{j}\right)+D_{N}\left({\tilde{A}}_{j}\right)=\zeta +\chi +\max\left\{\Gamma ,\zeta \right\},\forall j\in \left\{W+1,\ldots ,K\right\},$$

(58) $$D_{N,t,r_{q}}=\sum_{i\in B_{q}}D_{N}\left({\tilde{C}}_{i,r_{q}}\right)+D_{N}\left({\bar{A}}_{r_{q}}\right)+D_{N}\left({\tilde{A}}_{r_{q}}\right)=\left|B_{q}\right|\chi +\chi +\max\left\{\Gamma ,\zeta \right\},\forall q\in \left\{1,\ldots ,Q\right\}.$$

Now, we determine the minimum value for the parameter *T* (for which the interference alignment equations are satisfied) as follows:

(59) $$T=\max\left\{\max_{j\in \{1,\ldots ,K\}}\left\{d_{t,j}\right\},\max_{q\in \{1,\ldots ,Q\}}\left\{d_{t,r_{q}}\right\}\right\},$$

and from (53)–(59), we have

(60) $$\lim_{n\to \infty }\frac{T}{n^{K^{2}-K+QK}}=\chi +\max\left\{\Gamma ,\zeta \right\}+\max\left\{\max_{q\in \{1,\ldots ,Q\}}\left|B_{q}\right|\chi ,\zeta ,\Gamma \right\}.$$

However, we have:

$$\max_{q\in \{1,\ldots ,Q\}}\left|B_{q}\right|=\left\lceil \frac{W}{Q}\right\rceil ,$$

so we conclude that:

(61) $$\lim_{n\to \infty }\frac{T}{n^{K^{2}-K+QK}}=\chi +\max\left\{\Gamma ,\zeta \right\}+\max\left\{\left\lceil \frac{W}{Q}\right\rceil \chi ,\zeta ,\Gamma \right\}.$$

Up until now, we have considered any arbitrary real values for each parameter Γ,χ and ζ. Now, we make two additional assumptions for these parameters, which give us an achievable DoF. First, we set the normalized asymptotic dimension of the space at the clean receivers equal to that of the dirty receivers. Hence:

(62) Γ=ζ.

Second, we set the maximum normalized asymptotic dimension of the space at each MIMO C-IR receiving antenna to be less than or equal to that of the dirty receivers. Therefore, we have:

(63) $$\zeta \geq \left\lceil \frac{W}{Q}\right\rceil \chi .$$

Having (62) and (63), (61) will have the following form:

(64) $$\lim_{n\to \infty }\frac{T}{n^{K^{2}-K+QK}}=\chi +2\Gamma .$$

**Step 6: DoF Analysis**

Now, we characterize the total DoF. As stated before, we have *W* clean receivers, each with a normalized message dimension equal to Γ+χ, and K−W dirty receivers, each with a normalized message dimension equal to ζ (note that we set ζ=Γ).The total normalized transmission length is equal to χ+2Γ, so the total DoF has the following form:

(65) $$\mathrm{DoF}=\max_{\chi \geq 0,\Gamma \geq \left\lceil \frac{W}{Q}\right\rceil \chi }\frac{W(\chi +\Gamma )+(K-W)\Gamma }{\chi +2\Gamma },$$

and by assuming Γ=βχ, we have:

(66) $$\begin{array}{cc} \mathrm{DoF} & =\max_{\beta \geq \left\lceil \frac{W}{Q}\right\rceil }\frac{W(1+\beta )+(K-W)\beta }{1+2\beta } \end{array}$$

(67) $$\begin{array}{cc} \; & =\frac{K}{2}+\max_{\beta \geq \left\lceil \frac{W}{Q}\right\rceil }K\frac{\frac{W}{K}-\frac{1}{2}}{1+2\beta }=\frac{K}{2}+\max\left\{K\frac{\frac{W}{K}-\frac{1}{2}}{1+2\left\lceil \frac{W}{Q}\right\rceil },0\right\}. \end{array}$$

We remark that if $\frac{W}{K}>\frac{1}{2}$, we set $\beta =\left\lceil \frac{W}{Q}\right\rceil$, and if $\frac{W}{K}<\frac{1}{2}$, we tend β to *∞*. This completes the proof of the achievability of the first term of (11). The proof of the second term, i.e., min{Q,W}, is provided in Appendix A. □

**Remark** **2.** *It is known that the DoF is an appropriate performance metric that provides a capacity approximation accurate within o(log(ρ)) [1]. Therefore, Theorem 1 indicates that the approximate sum capacity of the K-user interference channel in the presence of a MIMO C-IR is lower bounded by $\left(\max\left\{\frac{K}{2}+\max\left\{0,K\frac{\frac{W}{K}-\frac{1}{2}}{1+2\left\lceil \frac{W}{Q}\right\rceil }\right\},\min\left\{Q,W\right\}\right\}-\epsilon \right)\log\left(1+\rho \right)+o\left(\log\left(\rho \right)\right),\forall \epsilon >0$. Now, we prove an improved achievable DoF for a special case of W and Q.*

**Theorem** **2.** *Assume W=QZ+P,P=1. Then, the achievable DoF (11) can be improved as follows:*

(68) $$\mathrm{DoF}=\max\left\{\frac{K}{2}+\max\left\{0,K\frac{\frac{W}{K}-\frac{1}{2}}{1+2\left\lfloor \frac{W}{Q}\right\rfloor }\right\},\min\left\{Q,W\right\}\right\}.$$

**Proof.**  The proof is provided in Appendix E.  □

**Remark** **3.** *Theorem 2 shows that the approximate sum capacity of the K-user interference channel with a MIMO C-IR is lower bounded by $\left(\max\left\{\frac{K}{2}+\max\left\{0,K\frac{\frac{W}{K}-\frac{1}{2}}{1+2\left\lfloor \frac{W}{Q}\right\rfloor }\right\},\min\left\{Q,W\right\}\right\}-\epsilon \right)$log(1+ρ)+o(log(ρ)),∀ϵ>0, where P=1 (we have W=QZ+P,0≤P<Q). From (11) and (68), we note that this lower bound is tighter than the previous bound.*

**Remark** **4.** *As expected, if we set Q=W=K, the maximum K DoF, which is the DoF at the absence of interference, is achievable for the MIMO C-IR.*

**Remark** **5.** *It was shown in [4] that an ordinary relay cannot increase the DoF of a K-user interference channel. The main difference here is that the instantaneity of the relay can significantly improve the DoF.*

**Remark** **6.** *Although we derived the achievable DoF for the asymptotic case, the achievability results are also valid for finite values of the auxiliary variable n, which determines the dimensions of beamforming vectors (see Equations (36)–(42)). Thus, if all interference alignment conditions (24)–(33) are satisfied and T is sufficiently large (as in Equation (59), i.e., larger than the sum of the interference and message subspaces), then for each receiver j∈{1,…,K}, there is the matrix $E_{j}$ such that if we multiply the vector of received signals in all frequency slots ($Y^{\left[j\right]}$) by $E_{j}$, the transmitted streams will be separated at each receiver with additive noise. Then, for the clean receivers j∈{1,…,W}, we have:*

(69) $$E_{j}Y^{\left[j\right]}=\left[\begin{array}{c} {\bar{x}}^{\left[j\right]}\\ {\tilde{x}}^{\left[j\right]} \end{array}\right]+{\hat{n}}^{\left[j\right]},$$

*where ${\hat{n}}^{\left[j\right]}$ is additive Gaussian noise, which is not necessarily white. Moreover, for the dirty receivers j∈{W+1,…,K}, we have:*

(70) $$E_{j}Y^{\left[j\right]}={\bar{x}}^{\left[j\right]}+{\hat{n}}^{\left[j\right]}.$$

*Thus, the proposed achievability scheme can be used for resource allocation problems, such as sum-rate optimization problems. This kind of utilization of interference alignment coding schemes for optimization problems was used in [35]. However, finding the optimal input distributions for the symbol streams ${\bar{x}}^{\left[i\right]}$ and ${\tilde{x}}^{\left[i\right]}$ and the optimal values for other parameters (t,s, and υ) in order to compare the performance of the proposed scheme with the performance of other signaling strategies (e.g., [36,37]) from the rate region perspective are still complicated problems and need complex optimization algorithms, which are directions for future research.*

Next, we introduce an upper bound for the sum DoF of the frequency-selective *K*-user interference channel assisted by the MIMO C-IR.

**Theorem** **3.** *Considering the functions $f^{\left[u,\omega_{t}\right]}$ to be linear in (3), the sum DoF of the frequency-selective K-user interference channel assisted by the MIMO C-IR can be upper-bounded as follows:*

(71) $$\sum_{i=1}^{K}d_{i}\leq \min\left\{\frac{K}{2}+\frac{WQ}{2(K-1)},K\right\}.$$

**Proof.**  By using (5)–(7), we have:

$$Y^{\left[j\right]}=\sum_{i=1}^{K}H^{\left[ji\right]}X^{\left[i\right]}+\sum_{u=1}^{W}H_{\mathrm{IR}-R}^{\left[ju\right]}\sum_{q=1}^{Q}A^{\left[uq\right]}\left(\sum_{i=1}^{K}H_{T-\mathrm{IR}}^{\left[qi\right]}X^{\left[i\right]}+Z_{\mathrm{IR}}^{\left[q\right]}\right)+Z^{\left[j\right]}$$

(72) $$=\sum_{i=1}^{K}\left(H^{\left[ji\right]}+\sum_{u=1}^{W}\sum_{q=1}^{Q}H_{\mathrm{IR}-R}^{\left[ju\right]}A^{\left[uq\right]}H_{T-\mathrm{IR}}^{\left[qi\right]}\right)X^{\left[i\right]}+{\hat{Z}}^{\left[j\right]}=\sum_{i=1}^{K}{\hat{H}}^{\left[ji\right]}X^{\left[i\right]}+{\hat{Z}}^{\left[j\right]},$$

where

(73) $${\hat{H}}^{\left[ji\right]}=H^{\left[ji\right]}+\sum_{u=1}^{W}\sum_{q=1}^{Q}H_{\mathrm{IR}-R}^{\left[ju\right]}A^{\left[uq\right]}H_{T-\mathrm{IR}}^{\left[qi\right]},$$

(74) $${\hat{Z}}^{\left[j\right]}=\sum_{u=1}^{W}\sum_{q=1}^{Q}H_{\mathrm{IR}-R}^{\left[ju\right]}A^{\left[uq\right]}Z_{\mathrm{IR}}^{\left[q\right]}+Z^{\left[j\right]}.$$

Now, consider the given i,j∈{1,…,K},i≠j. The matrices $A^{\left[uq\right]}$ must be chosen such that $\mathrm{rank}({\hat{H}}^{\left[ii\right]})=T,\forall i$; otherwise, the messages of each transmitter cannot be transmitted completely and the resulting upper bound for the sum DoF will decrease. For more clarity of the proof, we eliminate messages $w^{\left[k\right]},k\neq i,j$, and this causes the rates $r_{i}$ and $r_{j}$ to increase because of a data processing inequality [38] (Theorem 2.8.1). Hence, we have:

(75) $$Y^{\left[i\right]}={\hat{H}}^{\left[ii\right]}X^{\left[i\right]}+{\hat{H}}^{\left[ij\right]}X^{\left[j\right]}+{\hat{Z}}^{\left[i\right]},$$

(76) $$Y^{\left[j\right]}={\hat{H}}^{\left[ji\right]}X^{\left[i\right]}+{\hat{H}}^{\left[jj\right]}X^{\left[j\right]}+{\hat{Z}}^{\left[j\right]}.$$

Now, we define new variables as follows:

(77) $${Y^{\left[j\right]}}^{\prime }={\hat{H}}^{\left[ij\right]}{\left({\hat{H}}^{\left[jj\right]}\right)}^{-1}Y^{\left[j\right]}={\hat{H}}^{\left[ij\right]}{\left({\hat{H}}^{\left[jj\right]}\right)}^{-1}\left({\hat{H}}^{\left[ji\right]}X^{\left[i\right]}+{\hat{H}}^{\left[jj\right]}X^{\left[j\right]}\right)+{\hat{H}}^{\left[ij\right]}{\left({\hat{H}}^{\left[jj\right]}\right)}^{-1}{\hat{Z}}^{\left[j\right]},$$

(78) $${Y^{\left[j\right]}}^{\prime \prime }={\hat{H}}^{\left[ij\right]}{\left({\hat{H}}^{\left[jj\right]}\right)}^{-1}\left({\hat{H}}^{\left[ji\right]}X^{\left[i\right]}+{\hat{H}}^{\left[jj\right]}X^{\left[j\right]}\right)+{\hat{Z}}^{\left[i\right]}.$$

Then, we obtain:

(79) $$Tr_{i}\leq I\left(w^{\left[i\right]};Y^{\left[i\right]}\right)+\varepsilon ,$$

$$Tr_{j}\leq I\left(w^{\left[j\right]};Y^{\left[j\right]}\right)+\varepsilon \leq I\left(w^{\left[j\right]};Y^{\left[j\right]},{Y^{\left[j\right]}}^{\prime \prime }\right)+\varepsilon =I\left(w^{\left[j\right]};{Y^{\left[j\right]}}^{\prime \prime }\right)+I\left(w^{\left[j\right]};Y^{\left[j\right]}\left|{Y^{\left[j\right]}}^{\prime \prime }\right.\right)+\varepsilon$$

$$\leq I\left(w^{\left[j\right]};{Y^{\left[j\right]}}^{\prime \prime }\left|w^{\left[i\right]}\right.\right)+I\left(w^{\left[j\right]};Y^{\left[j\right]}\left|{Y^{\left[j\right]}}^{\prime \prime }\right.\right)+\varepsilon$$

(80) $$=I\left(w^{\left[j\right]};Y^{\left[i\right]}\left|w^{\left[i\right]}\right.\right)+I\left(w^{\left[j\right]};Y^{\left[j\right]}\left|{Y^{\left[j\right]}}^{\prime \prime }\right.\right)+\varepsilon .$$

Thus, we have:

(81) $$T\left(r_{i}+r_{j}\right)\leq I\left(w^{\left[i\right]},w^{\left[j\right]};Y^{\left[i\right]}\right)+I\left(w^{\left[j\right]};Y^{\left[j\right]}\left|{Y^{\left[j\right]}}^{\prime \prime }\right.\right)+2\varepsilon \leq \left(2T-R^{\left[ij\right]}\right)\log\left(1+\rho \right)+o\left(\log(\rho )\right),$$

where $R^{\left[ij\right]}=\mathrm{rank}\left({\hat{H}}^{\left[ij\right]}\right)$. By using the same argument, we obtain:

(82) $$r_{i}+r_{j}\leq \left(2-\frac{\max\left\{\mathrm{rank}\left({\hat{H}}^{\left[ij\right]}\right),\mathrm{rank}\left({\hat{H}}^{\left[ji\right]}\right)\right\}}{T}\right)\log\left(1+\rho \right)+o\left(\log(\rho )\right).$$

Therefore, we obtain:

$$\left(K-1\right)\sum_{i=1}^{K}r_{i}=\sum_{i\neq j}r_{i}+r_{j}$$

$$\leq \sum_{i\neq j}\left(2-\frac{\max\left\{\mathrm{rank}\left({\hat{H}}^{\left[ij\right]}\right),\mathrm{rank}\left({\hat{H}}^{\left[ji\right]}\right)\right\}}{T}\right)\log\left(1+\rho \right)+o\left(\log(\rho )\right)$$

(83) $$=\left(K\left(K-1\right)-\sum_{i\neq j}\left(\frac{\max\left\{\mathrm{rank}\left({\hat{H}}^{\left[ij\right]}\right),\mathrm{rank}\left({\hat{H}}^{\left[ji\right]}\right)\right\}}{T}\right)\right)\log\left(1+\rho \right)+o\left(\log(\rho )\right).$$

To minimize the term $\sum_{i\neq j}\left(\frac{\max\left\{\mathrm{rank}\left({\hat{H}}^{\left[ij\right]}\right),\mathrm{rank}\left({\hat{H}}^{\left[ji\right]}\right)\right\}}{T}\right)$, there are $WQT^{2}$ variables in the matrices $A^{\left[uq\right]}$. Every unit decrement of the rank of cross-link matrices requires *T* linear dependencies (*T* independent linear equations, which follow from the form of the arrangement of coefficients of equations); thus, we can see that:

(84) $$\sum_{i\neq j}\left(\frac{\max\left\{\mathrm{rank}\left({\hat{H}}^{\left[ij\right]}\right),\mathrm{rank}\left({\hat{H}}^{\left[ji\right]}\right)\right\}}{T}\right)\geq \frac{K(K-1)}{2}-\frac{WQ}{2}.$$

Considering (83) and (84), the upper bound (71) can be obtained. We note that $\sum_{i=1}^{K}d_{i}\leq K$ is obvious because of (79).  □

**Remark** **7.** *Theorem 3 indicates that the approximate sum capacity of the frequency-selective K-user interference channel assisted by the MIMO C-IR is upper-bounded by $\min\left\{\frac{K}{2}+\frac{WQ}{2(K-1)},K\right\}\log\left(1+\rho \right)+o\left(\log(\rho )\right)$.*

## 4. K-User Interference Channel in the Presence of NC-IR

In this section, we provide the lower and upper bounds for the sum DoF of the frequency-selective *K*-user interference channel in the presence of an NC-IR as follows.

**Theorem** **4.** *Consider $U,p,e,e^{\prime }\in W$ such that*

(85) $$U=pe+e^{\prime },0\leq e^{\prime }<p,\frac{K}{2}<U\leq K.$$

*Then, with an NC-IR with W=Q=pU antennas, the following DoF is achievable:*

(86) $$\mathrm{DoF}=\frac{K}{2}+\max\left\{K\frac{\frac{U}{K}-\frac{1}{2}}{1+2\left\lceil \frac{U}{p}\right\rceil },0\right\}.$$

**Proof.**  The proof is provided in Appendix F.  □

**Remark** **8.** *Theorem 4 indicates that the approximate sum capacity of a frequency-selective K-user interference channel in the presence of the NC-IR is lower bounded by $\left(\frac{K}{2}+\max\left\{K\frac{\frac{U}{K}-\frac{1}{2}}{1+2\left\lceil \frac{U}{p}\right\rceil },0\right\}-\epsilon \right)$log(1+ρ)+o(log(ρ)),∀ϵ>0.*

**Remark** **9.** *The active reconfigurable intelligent surface RIS can be modeled as a special case of an NC-IR [34]. It was proven in [34] that for an active RIS with Q=U(K−1)+U(K−U) antennas, the following DoF is achievable:*

(87) $$\mathrm{DoF}=\frac{K+U}{2},0\leq U\leq K.$$

*Therefore, we can see that for 0<Q<2(K−1), the achievable DoF (86) is dominant, and for Q≥2(K−1), the maximums of (86) and (87) form the maximum achievable DoF for the NC-IR.*

**Remark** **10.** *Considering Theorem 1, we can conclude that the maximum K DoF can be achieved by using Q=W=K antennas for a MIMO C-IR, but Q=K(K−1) antennas for achieving the maximum K DoF by an NC-IR is required, which grows quadratically and shows a loss of performance.*

Finally, we introduce an upper bound for the sum DoF of the frequency-selective *K*-user interference channel assisted by the NC-IR.

**Theorem** **5.** *Considering the functions $f^{\left[u,\omega_{t}\right]}$ to be linear in (4), the sum DoF of the frequency-selective K-user interference channel assisted by the NC-IR can be upper-bounded as follows:*

(88) $$\sum_{i=1}^{K}d_{i}\leq \min\left\{\frac{K}{2}+\frac{Q}{2(K-1)},K\right\}=\min\left\{\frac{K}{2}+\frac{W}{2(K-1)},K\right\}=\min\left\{\frac{K}{2}+\frac{\sqrt{WQ}}{2(K-1)},K\right\}.$$

**Proof.**  This theorem can be proven by using the same argument given for Theorem 3, except for the fact that the linear operation of the NC-IR can be represented as (8). Thus, matrices $A^{\left[u\right]}$ provide $QT^{2}$ variables, which changes (84) as follows:

(89) $$\sum_{i\neq j}\left(\frac{\max\left\{\mathrm{rank}\left({\hat{H}}^{\left[ij\right]}\right),\mathrm{rank}\left({\hat{H}}^{\left[ji\right]}\right)\right\}}{T}\right)\geq \frac{K(K-1)}{2}-\frac{Q}{2},$$

and which yields (88).  □

**Remark** **11.** *By considering Theorem 5, it can be seen that the approximate sum capacity of the frequency-selective K-user interference channel assisted by the NC-IR is upper-bounded by the expression $\min\left\{\frac{K}{2}+\frac{Q}{2(K-1)},K\right\}\log\left(1+\rho \right)+o\left(\log(\rho )\right)$.*

## 5. Numerical Results

**Figure 3:**
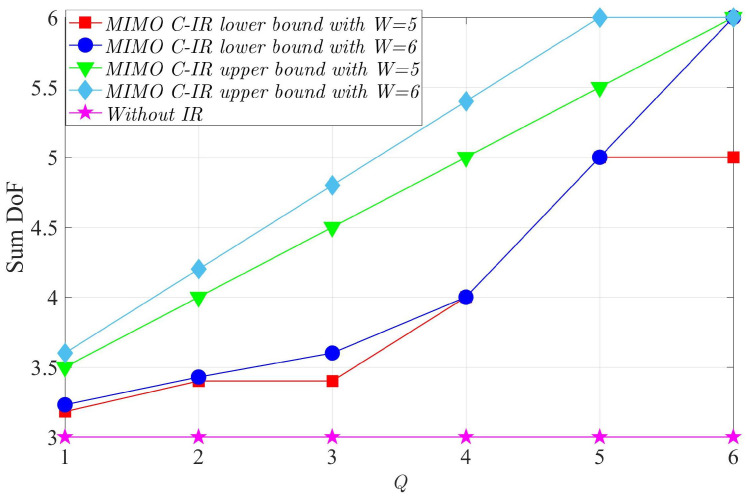
Comparison of lower and upper bounds for the sum DoF of the six-user interference channel in the presence of MIMO C-IR for the case without MIMO C-IR.

In this section, we numerically evaluate the lower and upper bounds for the sum DoF provided in the previous sections by using some examples. We note that the proposed bounds of the DoF of the MIMO C-IR and NC-IR and the existing bounds for the active RIS [31] (Theorems 1–5) do not depend on the distribution of channel coefficients, and the only required properties are independence and being drawn from a CDF, which is continuous. In Figure 3, we compare the lower and upper bounds for the sum DoF of a six-user interference channel in the presence of the MIMO C-IR for different values of *Q* and *W* and the case without the MIMO C-IR. We see that the achievable DoF can approach only a maximum value (K=6) when W=K=6. Additionally, we can observe that the maximum achieved DoF is equal to *W* when W≥4. Moreover, the maximum *K* DoFs can be achieved when Q=W.

**Figure 4:**
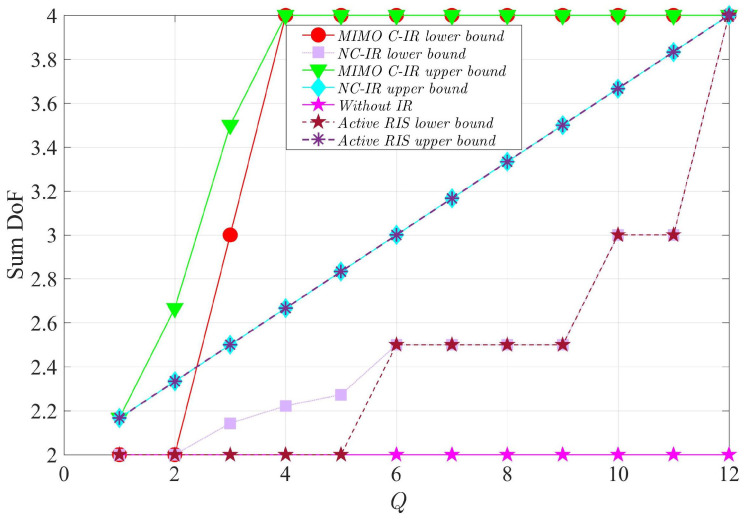
Comparison of lower and upper bounds for the sum DoF of the four-user interference channel in the presence of MIMO C-IR (with W=Q), NC-IR, active RIS and for the case without IR.

In Figure 4, we compare the lower and upper bounds for the sum DoF of four-user interference channels in the presence of the MIMO C-IR, NC-IR, and active RIS [34], and the case without an IR. We note that to have a fair comparison, we assume the same number of receiving and transmitting antennas for the MIMO C-IR (W=Q) as for the NC-IR and active RIS. These figures show that the maximum *K* DoF can be achieved by employing enough antennas for the MIMO C-IR, NC-IR, and active RIS. We see that the achievable DoF is considerably decreased for the NC-IR and active RIS, and this reduction is due to a lack of coordination between the antennas in the NC-IR and active RIS. Moreover, these figures show that the required number of antennas to allow the NC-IR and active RIS to achieve the maximum *K* DoF is quadratically larger than the required number of antennas for a MIMO C-IR, which shows a performance loss for the NC-IR due to a lack of coordination between the NC-IR antennas. In addition, the achievable DoF for the NC-IR is better than for the active RIS because the NC-IR can combine the received signals from different frequency slots (see Equation (4)); however, the model of the active RIS cannot conduct this operation.

**Figure 5:**
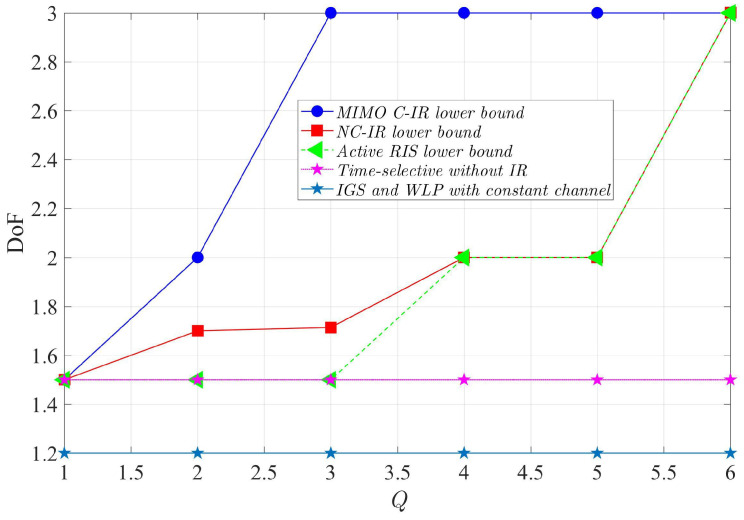
Comparison of the achievable sum DoF of the three-user interference channel in the presence of MIMO C-IR (with W=Q), NC-IR, and active RIS, the time-selective channel without IR [1], and the channel with constant coefficients using Improper Gaussian Signaling (IGS) [39] and Widely Linear Precoding (WLP) [40].

In Figure 5, we compare the achievable sum DoF of a three-user interference channel in the presence of the MIMO C-IR (with W=Q), NC-IR, and active RIS, a time-selective channel without an IR [1], and a channel with constant coefficients using Improper Gaussian Signaling (IGS) [39] and Widely Linear Precoding (WLP) [40]. We can see that the proposed scheme for the MIMO C-IR has the best performance and the IGS and WLP schemes for the constant channel have the worst performance.

## 6. Conclusions

In this paper, we studied the lower and upper bounds for the sum DoF of the IR-assisted frequency-selective *K*-user interference channel and proposed novel interference alignment-based coding schemes. The main novelty of this work is proposing a new interference alignment-based coding scheme in which receivers are partitioned into two groups called clean and dirty receivers. In this scheme, a part of the message streams of transmitters corresponding to clean receivers is de-multiplexed at the IR, and the IR uses these streams for an interference cancellation at the clean receivers, which causes an improvement of the DoF. This DoF improvement is achieved because in the interference alignment scheme, the dimension of interference subspaces decreases and the dimension of message subspaces increases at the clean receivers. For a MIMO C-IR, the antennas of which can have coordination with each other, and for an NC-IR (an IR with no coordination between the antennas), we derived achievable DoFs and observed a performance loss for the NC-IR compared with the MIMO C-IR. Moreover, we showed that by considering a number of antennas more than a finite value, a maximum *K* DoF is achievable for both the MIMO C-IR and NC-IR. The directions of our future work will contains the following aspects: (1) Finding tight bounds for the DoF of a time-selective *K*-user interference channel in the presence of an IR; (2) Extending our proposed coding scheme for more general wireless channels, e.g., an *X* network; (3) Extending our coding scheme to a scenario with an imperfect CSI.

## Appendix A

In this scheme, we use only one frequency slot: $\omega_{1}$. We set L=min{W,Q}. We assume that only the transmitters i∈{1,…,L} send their messages to the receivers j∈{1,…,L} via the symbols $X^{\left[i\right]}\left(\omega_{1}\right),i\in \left\{1,\ldots ,L\right\}$, and other transmitters are silent ($X^{\left[i\right]}\left(\omega_{1}\right)=0,\forall i\in \left\{L+1,\ldots ,K\right\}$). Considering (2), the MIMO C-IR can de-multiplex $X^{\left[i\right]}\left(\omega_{1}\right),\forall i\in \left\{1,\ldots ,L\right\}$ by using *L* linear equations in the first *L* receiving antennas almost surely because the matrix of the coefficients is in terms of independent random variables; thus, the matrix’s determinant is a non-zero polynomial of independent random variables with a continuous cumulative probability distribution, and considering [34] (Lemma 1), it is a non-zero with the probability 1. Then, the MIMO C-IR designs its transmitted signal to remove the interference in each receiver j∈{1,…,L} by solving the following linear equations:

(A1) $$-\sum_{i\in \{1,\ldots ,L\},i\neq j}H^{\left[ji\right]}\left(\omega_{1}\right)\left(X^{\left[i\right]}\left(\omega_{1}\right)+{\tilde{Z}}^{\left[i\right]}\left(\omega_{t}\right)\right)=\sum_{u=1}^{L}H_{\mathrm{IR}-R}^{\left[ju\right]}X_{\mathrm{IR}}^{\left[u\right]},\forall j\in \left\{1,\ldots ,L\right\},$$

(A2) $${\tilde{\tilde{Z}}}^{\left[j\right]}\left(\omega_{t}\right)=-\sum_{i\in \{1,\ldots ,L\},i\neq j}H^{\left[ji\right]}\left(\omega_{1}\right){\tilde{Z}}^{\left[i\right]}\left(\omega_{t}\right),$$

where ${\tilde{Z}}^{\left[i\right]}\left(\omega_{t}\right)$ is the detection noise for symbol $X^{\left[i\right]}\left(\omega_{t}\right)$ at the MIMO C-IR. Note that by using this procedure, the interference cancellation is conducted, but we have the additional noise ${\tilde{\tilde{Z}}}^{\left[j\right]}\left(\omega_{t}\right)$, which is negligible in a high signal to noise ratio (SNR) regime. Therefore, *L* symbols can be transmitted in one frequency slot, and the total *L* DoF is achievable. Thus, the second term in (11) is achievable, which completes the proof.

## Appendix B

Using (36) and (39), we characterize the message subspaces ${\bar{C}}_{j}$ and ${\tilde{C}}_{j}$ as follows:

$${\bar{C}}_{j}=\mathrm{span}\left(H^{\left[jj\right]}{\bar{V}}^{\left[j\right]}\right)=$$

(A3) $$\mathrm{span}\left\{H^{\left[jj\right]}\left[M(g_{1}\left(i,j\right),H^{\left[ji\right]},{\bar{S}}_{1})\right]\left[M(g_{2}\left(i,q\right),H_{T-\mathrm{IR}}^{\left[qi\right]},{\bar{S}}_{2})\right]w:g_{1}\in F\left({\bar{S}}_{1},\left\{1,\ldots ,n\right\}\right),g_{2}\in F\left({\bar{S}}_{2},\left\{1,\ldots ,sn\right\}\right)\right\},$$

where ${\bar{S}}_{1}$, ${\bar{S}}_{2}$, F(·,·), and M(·,·,·) are given by using (37), (38), (34), and (35), respectively.

$${\tilde{C}}_{j}=\mathrm{span}\left({\tilde{H}}^{\left[jj\right]}{\tilde{V}}^{\left[j\right]}\right)=$$

$$\mathrm{span}\left\{{\tilde{H}}^{\left[jj\right]}\left[M(g_{1}\left(i,j\right),{\tilde{H}}^{\left[ji\right]},{\bar{S}}_{1})\right]\left[M(g_{2}\left(i,q\right),H_{T-\mathrm{IR}}^{\left[qi\right]},{\tilde{S}}_{2})\right]\left[M(g_{3}\left(i,q\right),T^{\left[qi\right]},{\tilde{S}}_{3})\right]w:\right.$$

(A4) $$g_{1}\in F\left({\bar{S}}_{1},\left\{1,\ldots ,n\right\}\right),g_{2}\in F\left({\tilde{S}}_{2},\left\{1,\ldots ,sn\right\}\right)\left.,g_{3}\in F\left({\tilde{S}}_{3},\left\{1,\ldots ,\upsilon n\right\}\right)\right\},$$

where ${\tilde{S}}_{2}$ and ${\tilde{S}}_{3}$ are given by using (40) and (41), respectively.

To satisfy interference alignment Equation (24), the subspace ${\bar{A}}_{j}$ must be chosen such that:

$$\underset{i\in \{1,\ldots ,K\},i\neq j}{⋃}\left\{\mathrm{span}\left(H^{\left[ji\right]}{\bar{V}}^{\left[i\right]}\right)\right\}\subseteq {\bar{A}}_{j}.$$

Therefore, we characterize ${\bar{A}}_{j}$ as follows:

$${\bar{A}}_{j}=$$

(A5) $$\mathrm{span}\left\{\left[M(g_{1}\left(i,j\right),H^{\left[ji\right]},{\bar{S}}_{1})\right]\left[M(g_{2}\left(i,q\right),H_{T-\mathrm{IR}}^{\left[qi\right]},{\bar{S}}_{2})\right]w:g_{1}\in F\left({\bar{S}}_{1},\left\{1,\ldots ,n+1\right\}\right),g_{2}\in F\left({\bar{S}}_{2},\left\{1,\ldots ,n\max\left\{s,t\right\}\right\}\right)\right\},$$

where ${\bar{S}}_{1}$ and ${\bar{S}}_{2}$ are given by using (37) and (38), respectively. Note that to use the zero-forcing technique, the subspace of the interference must be a vector space, but the set of interference vectors, which is equal to $\underset{i\in \{1,\ldots ,K\},i\neq j}{⋃}\left\{\mathrm{span}\left(H^{\left[ji\right]}{\bar{V}}^{\left[i\right]}\right)\right\}$, is not a vector space; thus, we choose the subspace of interference (A5), which is easier to work with and includes $\underset{i\in \{1,\ldots ,K\},i\neq j}{⋃}\left\{\mathrm{span}\left(H^{\left[ji\right]}{\bar{V}}^{\left[i\right]}\right)\right\}$.

After that step, we analyze the dimension and the normalized asymptotic dimension of the messages and interference subspaces. First, we assume that the parameter *T* (the number of frequency slots) is sufficiently large, and at the end of Step 5 of the proof, we will choose the minimum value for *T* such that all message streams can be decodable and all interference alignment equations can be satisfied. Considering the natures of ${\bar{A}}_{j}$ in (A5), ${\bar{C}}_{j}$ in (A3), and ${\tilde{C}}_{j}$ in (A4), we can see from a statement of [34] (Lemma 2) that if we choose the variables $x_{k}$ as $H^{\left[ji\right]}\left(\omega_{t}\right),H_{T-\mathrm{IR}}^{\left[qi^{\prime }\right]}\left(\omega_{t}\right),i,i^{\prime },j\in \left\{1,\ldots ,K\right\},q\in \left\{1,\ldots ,Q\right\}$, $y_{k}$ as $H_{\mathrm{IR}-R}^{\left[ju\right]}\left(\omega_{t}\right),j\in \left\{W+1,\ldots ,K\right\},u\in \left\{1,\ldots ,W\right\}$, and $z_{k}$ as $H_{\mathrm{IR}-R}^{\left[ju\right]}\left(\omega_{t}\right),j\in \left\{1,\ldots ,W\right\},u\in \left\{1,\ldots ,W\right\}$, then by using [34] (Lemmas 1–3), the subspaces ${\bar{A}}_{j}$, ${\bar{C}}_{j}$ and ${\tilde{C}}_{j}$ are almost surely full-rank and linearly independent (all base vectors of these subspaces are linearly independent). In fact, if we take the constructing base vectors of ${\bar{A}}_{j}$, ${\bar{C}}_{j}$ and ${\tilde{C}}_{j}$ and construct a square matrix by choosing some rows of the matrix, we can see by using [34] (Lemmas 2–3) that the determinant of this square matrix will be a non-zero polynomial, and by using [34] (Lemma 1), it will be a non-zero with a probability equal to one; thus, all message streams are decodable at the clean receivers (by using zero forcing).

For more clarity, we will review [34] (Lemmas 1–3) as follows:

Ref. [34] (Lemma 1): Consider the *k* independent random variables $X_{1},\ldots ,X_{k}$, each constructed from a CDF, which is continuous. The probability of the event that the non-zero polynomial $P_{k}\left(X_{1},\ldots ,X_{k}\right)$, constructed from $X_{1},\ldots ,X_{k}$ with a finite degree, assumes the value zero is zero, i.e., $\mathrm{Pr}\{P_{k}\left(X_{1},\ldots ,X_{k}\right)=0\}=0$.

Ref. [34] (Lemma 2): Consider the three sets of variables $\left\{x_{i},i\in A_{x},\left|A_{x}\right|<\infty \right\}$, $\left\{y_{i},i\in A_{y},\left|A_{y}\right|<\infty \right\}$, and $\left\{z_{i},i\in A_{z},\left|A_{z}\right|<\infty \right\}$. Consider the following functions:

(A6) $$f_{j}=\prod_{i=1}^{\left|A_{x}\right|}{\left(x_{i}+\sum_{i^{\prime }\in C_{j},i^{\prime \prime }\in D_{j}}x_{i^{\prime }}y_{i^{\prime \prime }}{P_{1}}^{\left[i^{\prime }i^{\prime \prime }j\right]}\left(z_{k}:k\in A_{z}\right)+y_{i^{\prime \prime }}{P_{2}}^{\left[i^{\prime }i^{\prime \prime }j\right]}\left(z_{k}:k\in A_{z}\right)\right)}^{a_{i}^{j}},$$

$$\left(a_{1}^{j},\ldots ,a_{\left|A_{x}\right|}^{j}\right)\in W^{\left|A_{x}\right|},j\in \left\{1,\ldots ,J\right\},$$

where $P_{1}^{\left[i^{\prime }i^{\prime \prime }j\right]}\left(\cdot \right)$ and $P_{2}^{\left[i^{\prime }i^{\prime \prime }j\right]}\left(\cdot \right)$ are fractional polynomials and for ∀j, we have $\left|C_{j}\right|,\left|D_{j}\right|<\infty$. If for $\forall j,j^{\prime }$ with $j\neq j^{\prime }$, $\left(a_{1}^{j},\ldots ,a_{\left|A_{x}\right|}^{j}\right)\neq \left(a_{1}^{j^{\prime }},\ldots ,a_{\left|A_{x}\right|}^{j^{\prime }}\right)$, then the functions $f_{j}$ will be linearly independent.

Ref. [34] (Lemma 3): Consider the set of non-zero linearly independent fractional polynomials $\left\{P^{\left[j\right]}\left(\cdot \right),j\in \left\{1,\ldots ,J\right\}\right\}$ and consider the *J* sets of variables $X_{j}=\{x_{i}^{j}:i\in I,I\subseteq N,|I|<\infty \}$, j∈{1,…,J}. The determinant of the following matrix will be a non-zero fractional polynomial:

(A7) $$A=\left[\begin{array}{cccc} P^{\left[1\right]}\left(X_{1}\right) & P^{\left[2\right]}\left(X_{1}\right) & \cdots & P^{\left[J\right]}\left(X_{1}\right)\\ P^{\left[1\right]}\left(X_{2}\right) & P^{\left[2\right]}\left(X_{2}\right) & \cdots & P^{\left[J\right]}\left(X_{2}\right)\\ \vdots & \vdots & \ddots & \vdots \\ P^{\left[1\right]}\left(X_{J}\right) & P^{\left[2\right]}\left(X_{J}\right) & \cdots & P^{\left[J\right]}\left(X_{J}\right) \end{array}\right].$$

Now, we have to make sure that interference alignment Equations (24) and (25) are satisfied by analyzing the dimension of message streams and interference. The dimension of the message subspaces ${\bar{C}}_{j}$ and ${\tilde{C}}_{j}$, which is equal to the number of its base vectors in (A3) and (A4), can be characterized as follows:

(A8) $$d\left({\bar{C}}_{j}\right)=n^{K^{2}-K}{\left(sn\right)}^{QK},$$

(A9) $$d\left({\tilde{C}}_{j}\right)=n^{K^{2}-K}{\left(sn\right)}^{\varphi }{\left(\upsilon n\right)}^{\theta },$$

where

$$\varphi =\sum_{q^{\prime }=1}^{Q}\left(K-\left|B_{q^{\prime }}\right|\right)=KQ-\sum_{q^{\prime }=1}^{Q}\left|B_{q^{\prime }}\right|=KQ-W,$$

$$\theta =\sum_{q^{\prime }=1}^{Q}\left|B_{q^{\prime }}\right|=W.$$

The dimension of the interference subspace ${\bar{A}}_{j}$, which is equal to the number of its base vectors in (A5), is:

(A10) $$d\left({\bar{A}}_{j}\right)={\left(n+1\right)}^{K^{2}-K}{\left(\max\{sn,tn\}\right)}^{QK}.$$

We can see from (A8)–(A10) and (10) that $l=K^{2}-K+QK$. We define the following parameters:

(A11) $$\Gamma =s^{QK},$$

(A12) $$\chi =s^{QK-W}\upsilon^{W},$$

(A13) $$\zeta =t^{QK}.$$

Considering (A8)–(A13) and (10), the normalized asymptotic dimensions of the message and interference subspaces are:

(A14) $$D_{N}\left({\bar{C}}_{j}\right)=\Gamma ,$$

(A15) $$D_{N}\left({\tilde{C}}_{j}\right)=\chi ,$$

(A16) $$D_{N}\left({\bar{A}}_{j}\right)=\max\left\{\Gamma ,\zeta \right\}.$$

Interference alignment Equations (24) and (25) are satisfied because we can see that the normalized asymptotic dimension of the interference induced by ${\bar{V}}^{\left[i\right]}{\bar{x}}^{\left[i\right]},i\in \left\{1,\ldots ,W\right\},i\neq j$ is Γ and the normalized asymptotic dimension of the interference induced by ${\bar{V}}^{\left[i\right]}{\bar{x}}^{\left[i\right]},i\in \left\{W+1,\ldots ,K\right\}$ is ζ.

## Appendix C

Using (42), we can characterize the message subspace ${\bar{C}}_{j}$ as follows:

$${\bar{C}}_{j}=\mathrm{span}\left(H^{\left[jj\right]}{\bar{V}}^{\left[j\right]}\right)=$$

(A17) $$\mathrm{span}\left\{H^{\left[jj\right]}\left[M(g_{1}\left(i,j\right),H^{\left[ji\right]},{\bar{S}}_{1})\right]\left[M(g_{2}\left(i,q\right),H_{T-\mathrm{IR}}^{\left[qi\right]},{\bar{S}}_{2})\right]w:g_{1}\in F\left({\bar{S}}_{1},\left\{1,\ldots ,n\right\}\right),g_{2}\in F\left({\bar{S}}_{2},\left\{1,\ldots ,tn\right\}\right)\right\},$$

where ${\bar{S}}_{1}$, ${\bar{S}}_{2}$, F(·,·), and M(·,·,·) are given by using (37), (38), (34), and (35), respectively.

To satisfy interference alignment Equation (26), the subspace ${\bar{A}}_{j}$ must be chosen such that:

$$\underset{i\in \{1,\ldots ,K\},i\neq j}{⋃}\left\{\mathrm{span}\left(H^{\left[ji\right]}{\bar{V}}^{\left[i\right]}\right)\right\}\subseteq {\bar{A}}_{j}.$$

Therefore, we characterize ${\bar{A}}_{j}$ as follows:

$${\bar{A}}_{j}=$$

(A18) $$\mathrm{span}\left\{\left[M(g_{1}\left(i,j\right),H^{\left[ji\right]},{\bar{S}}_{1})\right]\left[M(g_{2}\left(i,q\right),H_{T-\mathrm{IR}}^{\left[qi\right]},{\bar{S}}_{2})\right]w:g_{1}\in F\left({\bar{S}}_{1},\left\{1,\ldots ,n+1\right\}\right),g_{2}\in F\left({\bar{S}}_{2},\left\{1,\ldots ,n\max\left\{s,t\right\}\right\}\right)\right\},$$

where ${\bar{S}}_{1}$ and ${\bar{S}}_{2}$ are given by (37) and (38), respectively. To satisfy interference alignment Equation (28), the subspace ${\tilde{A}}_{j}$ must be chosen such that:

$$\underset{i\in \{1,\ldots ,W\}}{⋃}\left\{\mathrm{span}\left({\tilde{H}}^{\left[ji\right]}{\tilde{V}}^{\left[i\right]}\right)\right\}\subseteq {\tilde{A}}_{j}.$$

Therefore, we characterize subspace ${\bar{A}}_{j}$ as follows:

$${\tilde{A}}_{j}=\mathrm{span}\left\{\left[M(g_{1}\left(i,j\right),{\tilde{H}}^{\left[ji\right]},{\bar{S}}_{1})\right]\left[M(g_{2}\left(i,q\right),H_{T-\mathrm{IR}}^{\left[qi\right]},{\tilde{S}}_{2})\right]\left[M(g_{3}\left(i,q\right),T^{\left[qi\right]},{\tilde{S}}_{3})\right]w:\right.$$

(A19) $$g_{1}\in F\left({\bar{S}}_{1},\left\{1,\ldots ,n+1\right\}\right),g_{2}\in F\left({\tilde{S}}_{2},\left\{1,\ldots ,sn\right\}\right)\left.,g_{3}\in F\left({\tilde{S}}_{3},\left\{1,\ldots ,\upsilon n\right\}\right)\right\},$$

where ${\tilde{S}}_{2}$ and ${\tilde{S}}_{3}$ are given by using (40) and (41), respectively.

By using the same argument given for the clean receivers, subspaces ${\bar{A}}_{j}$, ${\tilde{A}}_{j}$, and ${\bar{C}}_{j}$ are full-rank and linearly independent almost surely, i.e., all base vectors of these subspaces are linearly independent. Now, we analyze the dimensions of the message and interference subspaces. By calculating the number of base vectors of the message subspace ${\bar{C}}_{j}$ in (A17), we have:

(A20) $$d\left({\bar{C}}_{j}\right)=n^{K^{2}-K}{\left(tn\right)}^{QK},$$

$$D_{N}\left({\bar{C}}_{j}\right)=\zeta ,$$

and for the interference subspaces in (A18) and (A19), we have:

(A21) $$d\left({\bar{A}}_{j}\right)={\left(n+1\right)}^{K^{2}-K}{\left(\max\{sn,tn\}\right)}^{QK},$$

$$D_{N}\left({\bar{A}}_{j}\right)=\max\left\{\Gamma ,\zeta \right\},$$

(A22) $$d\left({\tilde{A}}_{j}\right)={\left(n+1\right)}^{K^{2}-K}{\left(sn\right)}^{QK-W}{\left(\upsilon n\right)}^{W},$$

$$D_{N}\left({\tilde{A}}_{j}\right)=\chi .$$

Therefore, we can see that interference alignment Equations (26)–(29) are satisfied because the normalized asymptotic dimension of the interference subspace induced by ${\tilde{V}}^{\left[i\right]}{\tilde{x}}^{\left[i\right]},i\in \left\{1,..,W\right\}$ is χ, the normalized asymptotic dimension of the interference subspace induced by ${\bar{V}}^{\left[i\right]}{\bar{x}}^{\left[i\right]},i\in \left\{1,..,W\right\}$ is Γ, and the normalized asymptotic dimension of the interference subspace induced by ${\bar{V}}^{\left[i\right]}{\bar{x}}^{\left[i\right]},i\in \left\{W+1,..,K\right\},i\neq j$ is ζ.

## Appendix D

Using (39), we can characterize the message subspaces ${\tilde{C}}_{i,r_{q}},i\in B_{q}$ as follows:

$${\tilde{C}}_{i,r_{q}}=\mathrm{span}\left(H_{T-\mathrm{IR}}^{\left[qi\right]}{\tilde{V}}^{\left[i\right]}\right)=$$

$$\mathrm{span}\left\{H_{T-\mathrm{IR}}^{\left[qi\right]}\left[M(g_{1}\left(i,j\right),{\tilde{H}}^{\left[ji\right]},{\bar{S}}_{1})\right]\left[M(g_{2}\left(i,q\right),H_{T-\mathrm{IR}}^{\left[qi\right]},{\tilde{S}}_{2})\right]\left[M(g_{3}\left(i,q\right),T^{\left[qi\right]},{\tilde{S}}_{3})\right]w:\right.$$

(A23) $$g_{1}\in F\left({\bar{S}}_{1},\left\{1,\ldots ,n\right\}\right),g_{2}\in F\left({\tilde{S}}_{2},\left\{1,\ldots ,sn\right\}\right)\left.,g_{3}\in F\left({\tilde{S}}_{3},\left\{1,\ldots ,\upsilon n\right\}\right)\right\},$$

where ${\tilde{S}}_{2}$, ${\tilde{S}}_{3}$, F(·,·), and M(·,·,·) are given by using (40), (41), (34), and (35), respectively.

To satisfy interference alignment Equation (30), the subspace ${\bar{A}}_{r_{q}}$ must be chosen such that:

$$\underset{i\in \{1,\ldots ,K\}}{⋃}\left\{\mathrm{span}\left(H_{T-\mathrm{IR}}^{\left[qi\right]}{\bar{V}}^{\left[i\right]}\right)\right\}\subseteq {\bar{A}}_{r_{q}}.$$

Therefore, we can characterize ${\bar{A}}_{j}$ as follows:

$${\bar{A}}_{r_{q}}=$$

(A24) $$\mathrm{span}\left\{\left[M(g_{1}\left(i,j\right),H^{\left[ji\right]},{\bar{S}}_{1})\right]\left[M(g_{2}\left(i,q\right),H_{T-\mathrm{IR}}^{\left[qi\right]},{\bar{S}}_{2})\right]w:g_{1}\in F\left({\bar{S}}_{1},\left\{1,\ldots ,n\right\}\right),g_{2}\in F\left({\bar{S}}_{2},\left\{1,\ldots ,n\max\left\{s,t\right\}+1\right\}\right)\right\},$$

where ${\bar{S}}_{1}$ and ${\bar{S}}_{2}$ are given by using (37) and (38), respectively.

To satisfy interference alignment Equation (32), the subspace ${\tilde{A}}_{r_{q}}$ must be chosen such that:

$$\underset{i\in \left\{1,\ldots ,W\right\},i\notin B_{q}}{⋃}\left\{\mathrm{span}\left(H_{T-\mathrm{IR}}^{\left[qi\right]}{\tilde{V}}^{\left[i\right]}\right)\right\}\subseteq {\tilde{A}}_{r_{q}}.$$

Therefore, we characterize ${\tilde{A}}_{j}$ as follows:

$${\tilde{A}}_{r_{q}}=\mathrm{span}\left\{\left[M(g_{1}\left(i,j\right),{\tilde{H}}^{\left[ji\right]},{\bar{S}}_{1})\right]\left[M(g_{2}\left(i,q\right),H_{T-\mathrm{IR}}^{\left[qi\right]},{\tilde{S}}_{2})\right]\left[M(g_{3}\left(i,q\right),T^{\left[qi\right]},{\tilde{S}}_{3})\right]w:\right.$$

(A25) $$g_{1}\in F\left({\bar{S}}_{1},\left\{1,\ldots ,n\right\}\right),g_{2}\in F\left({\tilde{S}}_{2},\left\{1,\ldots ,sn+1\right\}\right)\left.,g_{3}\in F\left({\tilde{S}}_{3},\left\{1,\ldots ,\upsilon n\right\}\right)\right\},$$

where ${\tilde{S}}_{2}$ and ${\tilde{S}}_{3}$ are given by using (40) and (41), respectively.

By using the same argument given for the clean receivers, subspaces ${\bar{A}}_{r_{q}}$, ${\tilde{A}}_{r_{q}}$ and ${\tilde{C}}_{i,r_{q}},i\in B_{q}$ are full-rank and linearly independent almost surely, i.e., all base vectors of these subspaces are linearly independent. Now, by calculating the number of base vectors, we can analyze the dimensions of the subspaces ${\tilde{C}}_{i,r_{q}},i\in B_{q}$, ${\bar{A}}_{r_{q}}$ and ${\tilde{A}}_{r_{q}}$:

(A26) $$d\left({\tilde{C}}_{i,r_{q}}\right)=n^{K^{2}-K}{\left(sn\right)}^{QK-W}{\left(\upsilon n\right)}^{W},\forall i\in B_{q},$$

$$D_{N}\left({\tilde{C}}_{i,r_{q}}\right)=\chi .$$

Thus, the normalized dimension of the total subspaces, the message symbols of which may be de-multiplexed (${\tilde{x}}^{\left[i\right]},i\in B_{q}$) at the MIMO C-IR *q*-th receiving antenna is:

$$\sum_{i\in B_{q}}D_{N}\left({\tilde{C}}_{i,r_{q}}\right)=\left|B_{q}\right|\chi .$$

For ${\bar{A}}_{r_{q}}$, we have:

(A27) $$d\left({\bar{A}}_{r_{q}}\right)=n^{K^{2}-K}{\left(\max\{sn,tn\}+1\right)}^{KQ},$$

$$D_{N}\left({\bar{A}}_{r_{q}}\right)=\max\left\{\Gamma ,\zeta \right\},$$

and for ${\tilde{A}}_{r_{q}}$, we have:

(A28) $$d\left({\tilde{A}}_{r_{q}}\right)=n^{K^{2}-K}{\left(sn+1\right)}^{QK-W}{\left(\upsilon n\right)}^{W},$$

$$D_{N}\left({\tilde{A}}_{r_{q}}\right)=\chi .$$

Thus, we can see that interference alignment Equations (30)–(33) are satisfied.

## Appendix E

The second term of (68) is exactly the same as the second term of (11) in Theorem 1. The proof of the first term is similar to the proof of the first term of (11) in Theorem 1 with a difference in the MIMO C-IR de-multiplexing method. In the proof of Theorem 1, each MIMO C-IR receiving antenna *q* de-multiplexes the message streams ${\tilde{x}}_{i},i\in B_{q}$ separately without a coordination with other receiving antennas. However, in the proof of this theorem, we use a coordination between the MIMO C-IR receiving antennas. Without a loss of generality, assume that $\left|B_{1}\right|=Z+1$ and $\left|B_{q}\right|=Z,q\neq 1$. To de-multiplex the message streams ${\tilde{x}}_{i},i\in \left\{1,\ldots ,W\right\}$ at the MIMO C-IR, first we de-multiplex the message streams ${\tilde{x}}_{i},i\in B_{q},q\neq 1$ at the *q*-th MIMO C-IR receiving antenna separately. Then, to de-multiplex the message streams ${\tilde{x}}_{i},i\in B_{1}$, we first remove the interference induced by the message streams ${\tilde{x}}_{i},i\in \left\{1,\ldots ,W\right\},i\notin B_{1}$. This results in a decrement in the total normalized asymptotic dimension at the first receiving antenna of the MIMO C-IR (the amount of decrement is χ), so (58) changes into the following form for q=1:

(A29) $$D_{N,t,r_{1}}=\left\lfloor \frac{W}{Q}\right\rfloor \chi +\chi +\max\left\{\Gamma ,\zeta \right\},$$

and the constraint (63) changes into the following form:

(A30) $$\zeta \geq \left\lfloor \frac{W}{Q}\right\rfloor \chi .$$

Then, we see that the DoF (68) is achievable.

## Appendix F

The proof of this theorem is similar to the first term in the proof of Theorem 1. Here, we use the variable *U* introduced in the statement of the theorem to denote the number of clean receivers. Note that to avoid several notations, we use the same notations (such as the name of sets and vector subspaces) used in the proof of Theorem 1. Thus, from now on, these notations belong to this theorem. Our proof has six steps as follows.

**Step 1: Dividing Receivers, Transmitters, and NC-IR Antennas**

Using the same method as Step 1 of the proof of the first term in Theorem 1, we divide the transmitters into two partitions. For the transmitters i∈{1,…,U}, we provide two sets of symbol streams: ${\bar{x}}^{\left[i\right]}$ and ${\tilde{x}}^{\left[i\right]}$. The matrices ${\bar{V}}^{\left[i\right]}$ and ${\tilde{V}}^{\left[i\right]}$ are beamforming matrices, the columns of which are the beamforming vectors for each element of ${\bar{x}}^{\left[i\right]}$ and ${\tilde{x}}^{\left[i\right]}$, respectively. For the transmitters i∈{U+1,…,K}, we provide only one set of the symbol stream ${\bar{x}}^{\left[i\right]}$, and the matrix ${\bar{V}}^{\left[i\right]}$ is the beamforming matrix for the symbols ${\bar{x}}^{\left[i\right]}$. Hence, the vectors $X^{\left[i\right]}$ will have the forms of (12) and (13) by using the setting W=U. The reason for this kind of partitioning is the same as in Theorem 1. The main difference here is in the interference alignment scheme used for de-multiplexing the message streams ${\tilde{x}}^{\left[i\right]},i\in \left\{1,\ldots ,U\right\}$ in the NC-IR receiving antennas.

Next, we divide the transmitters i∈{1,…,U} into the *p* distinct sets $E_{l},l\in \left\{1,\ldots ,p\right\}$ such that for $l\in \{1,\ldots ,e^{\prime }\}$, we have $\left|E_{l}\right|=e+1$, and for $l\in \{e^{\prime }+1,\ldots ,p\}$, we have $\left|E_{l}\right|=e$. Similarly, we divide the NC-IR antennas into the *p* distinct sets $F_{l},l\in \left\{1,\ldots ,p\right\}$ such that $\left|F_{l}\right|=U,\forall l\in \left\{1,\ldots ,p\right\}$. Now, we design the beamforming matrices ${\bar{V}}^{\left[i\right]}$ and ${\tilde{V}}^{\left[i\right]}$ such that the message streams ${\tilde{x}}^{\left[i\right]},i\in E_{l}$ may be de-multiplexed in each of the NC-IR antennas $u\in F_{l}$ for ∀l∈{1,…,p}.

**Step 2: Interference Cancellation at the Clean Receivers and Equivalent Channel at the Dirty Receivers**

For the interference cancellation, we design the outputs of antennas in the set $F_{l}$ such that the interference induced by the message streams ${\tilde{x}}^{\left[i\right]},i\in E_{l}$ is removed at the clean receivers j∈{1,…,U}. Thus, the NC-IR antennas’ transmitted signal must be designed such that they satisfy the following:

(A31) $$-\sum_{i\in E_{l},i\neq j}H^{\left[ji\right]}{\tilde{V}}^{\left[i\right]}{\tilde{x}}^{\left[i\right]}=\sum_{u\in F_{l}}H_{\mathrm{IR}-R}^{\left[ju\right]}X_{\mathrm{IR}}^{\left[u\right]},\forall j\in \left\{1,\ldots ,U\right\},\forall l\in \left\{1,\ldots ,p\right\}.$$

The solution to (A31) can be derived as follows:

(A32) $$X_{\mathrm{IR}}^{\left[u\right]}=\sum_{j\in \{1,\ldots ,U\}}\sum_{i\in E_{l},i\neq j}H_{\mathrm{inv}}^{\left[ju\right]}H^{\left[ji\right]}{\tilde{V}}^{\left[ji\right]}{\tilde{x}}^{\left[i\right]},\forall u\in F_{l},$$

where $H_{\mathrm{inv}}^{\left[ju\right]}$ is a T×T diagonal matrix and its *t*-th diagonal element is a fractional polynomial in terms of $H_{\mathrm{IR}-R}^{\left[j^{\prime }u^{\prime }\right]}\left(\omega_{t}\right),u^{\prime }\in F_{l},j^{\prime }\in \left\{1,\ldots ,U\right\}$. This solution exists almost surely because the matrix of the coefficients of the linear equations is in terms of independent random variables, its determinant is a non-zero polynomial in terms of these random variables drawn from a CDF, which is continuous, and by using [34] (Lemma 1), it is a non-zero with the probability 1. Note that each NC-IR receiving antenna de-multiplexes the symbol streams ${\tilde{x}}^{\left[i\right]}$ with additive noise. This event does not disturb the equations above because if each symbol is replaced by a symbol with additive noise, the interference cancellation holds but we have additional noise, which is negligible in a high SNR regime. We can see that the received signals at the receivers have the same forms as (22) and $H_{\mathrm{inv}}^{\left[ju\right]}$ and the equivalent channel matrix ${\tilde{H}}^{\left[ji\right]}$ has the same properties introduced in Step 2 of the proof of the first term in Theorem 1.

**Step 3: Interference Alignment Equations**

The interference alignment equations and message and interference subspaces for the clean and dirty receivers are the same as in Step 3 of the proof of the first term in Theorem 1 ((24)–(29)) if we replace *W* with *U*. Consider q∈{1,…,pU}: we define the function L(q)=l if $q\in F_{l}$ (*l* is unique because the sets $F_{l}$ are disjointed). We designed the interference alignment scheme such that the symbol streams ${\tilde{x}}^{\left[i\right]},i\in E_{L(q)}$ can be de-multiplexed at the *q*-th receiving antenna of the NC-IR. Thus, the interference alignment equations for the NC-IR change as follows.

To this end, all the interference induced by the symbol streams ${\bar{x}}^{\left[i\right]}$ must align into a limited subspace. Therefore, at the *q*-th receiving antenna of the NC-IR and for each i∈{1,…,K}, we must have:

(A33) $$\mathrm{span}\left(H_{T-\mathrm{IR}}^{\left[qi\right]}{\bar{V}}^{\left[i\right]}\right)\subseteq {\bar{A}}_{r_{q}},$$

where ${\bar{A}}_{r_{q}}$ is considered a subspace for which we have:

(A34) $$\max_{i\in \{1,\ldots ,K\}}D_{N}\left(\mathrm{span}\left(H_{T-\mathrm{IR}}^{\left[qi\right]}{\bar{V}}^{\left[i\right]}\right)\right)=D_{N}\left({\bar{A}}_{r_{q}}\right).$$

Then, for each $i\in \left\{1,\ldots ,U\right\},i\notin E_{L(q)}$, we have:

(A35) $$\mathrm{span}\left(H_{T-\mathrm{IR}}^{\left[qi\right]}{\tilde{V}}^{\left[i\right]}\right)\subseteq {\tilde{A}}_{r_{q}},$$

where ${\tilde{A}}_{r_{q}}$ is considered a subspace for which we have:

(A36) $$\max_{i\in \left\{1,\ldots ,U\right\},i\notin E_{L(q)}}D_{N}\left(\mathrm{span}\left(H_{T-\mathrm{IR}}^{\left[qi\right]}{\tilde{V}}^{\left[i\right]}\right)\right)=D_{N}\left({\tilde{A}}_{r_{q}}\right).$$

Moreover, we define ${\tilde{C}}_{i,r_{q}},i\in E_{L(q)}$ as the message subspaces, which can be de-multiplexed at the NC-IR *q*-th antenna as follows:

(A37) $${\tilde{C}}_{i,r_{q}}=\mathrm{span}\left(H_{T-\mathrm{IR}}^{\left[qi\right]}{\tilde{V}}^{\left[i\right]}\right),i\in E_{L(q)}.$$

We want ${\tilde{C}}_{i,r_{q}},\forall i\in E_{L(q)}$, ${\bar{A}}_{r_{q}}$ and ${\tilde{A}}_{r_{q}}$ to be full-rank and linearly independent, so we can make sure that the message streams ${\tilde{x}}^{\left[i\right]},i\in E_{L(q)}$ can be de-multiplexed at the *q*-th NC-IR antenna. In Steps 4 and 5, we prove the existence of such beamforming vectors, messages, and interference subspaces, which satisfies the previous interference alignment equations for the clean and dirty receivers and the MIMO C-IR. In Step 6, we analyze the achieved DoF by using the beamforming vectors’ design.

**Step 4: Beamforming Matrix Design**

The beamforming matrices ${\bar{V}}^{\left[i\right]},\forall i\in \left\{1,\ldots ,K\right\}$ are the same as (36) and (42) if we replace *W* with *U*. For ${\tilde{V}}^{\left[i\right]}$, we have:

(A38) $${\tilde{V}}^{\left[i\right]}=\left\{\left[M(g_{1}\left(i,j\right),{\tilde{H}}^{\left[ji\right]},{\bar{S}}_{1})\right]\left[M(g_{2}\left(i,q\right),H_{T-\mathrm{IR}}^{\left[qi\right]},{\tilde{S}}_{2})\right]\left[M(g_{3}\left(i,q\right),T^{\left[qi\right]},{\tilde{S}}_{3})\right]w:\right.$$

(A39) $$g_{1}\in F\left({\bar{S}}_{1},\left\{1,\ldots ,n\right\}\right),g_{2}\in F\left({\tilde{S}}_{2},\left\{1,\ldots ,sn\right\}\right)\left.,g_{3}\in F\left({\tilde{S}}_{3},\left\{1,\ldots ,\upsilon n\right\}\right)\right\},$$

where ${\bar{S}}_{1}$, F(·,·) and M(·,·,·) are given by using (37), (34), and (35), respectively, and we have:

(A40) $${\tilde{S}}_{2}=\left\{\left(i,q\right)\left|i\in \left\{1,\ldots ,K\right\},i\notin E_{L(q)},q\in \left\{1,\ldots ,Q\right\}\right.\right\},$$

(A41) $${\tilde{S}}_{3}=\left\{\left(i,q\right)\left|i\in E_{L(q)},q\in \left\{1,\ldots ,Q\right\}\right.\right\}.$$

$T^{\left[q^{\prime \prime }i^{\prime \prime \prime }\right]}$s are T×T diagonal random matrices for each (i,q), where each diagonal element for each matrix is drawn independently and its CDF is continuous.

Note that similar to the proof of Theorem 1, each value of the parameters s,υ and *t* can be approximated by using rational numbers with arbitrarily small errors, and by choosing a sufficiently large *n*, the parameters sn,υn and tn will be integers.

**Step 5: Validity of Interference Alignment Conditions and Decodability of Message Symbols**

*(1) Validity of Interference Alignment Conditions at Clean Receivers j∈{1,…,U}:* The message subspace ${\bar{C}}_{j}$ and the interference subspace ${\bar{A}}_{j}$ will be exactly the same as (A3) and (A5). The message subspaces ${\tilde{C}}_{j}$ will change as follows:

$${\tilde{C}}_{j}=\mathrm{span}\left({\tilde{H}}^{\left[jj\right]}{\tilde{V}}^{\left[j\right]}\right)=$$

$$\mathrm{span}\left\{{\tilde{H}}^{\left[jj\right]}\left[M(g_{1}\left(i,j\right),{\tilde{H}}^{\left[ji\right]},{\bar{S}}_{1})\right]\left[M(g_{2}\left(i,q\right),H_{T-\mathrm{IR}}^{\left[qi\right]},{\tilde{S}}_{2})\right]\left[M(g_{3}\left(i,q\right),T^{\left[qi\right]},{\tilde{S}}_{3})\right]w:\right.$$

(A42) $$g_{1}\in F\left({\bar{S}}_{1},\left\{1,\ldots ,n\right\}\right),g_{2}\in F\left({\tilde{S}}_{2},\left\{1,\ldots ,sn\right\}\right)\left.,g_{3}\in F\left({\tilde{S}}_{3},\left\{1,\ldots ,\upsilon n\right\}\right)\right\},$$

where ${\tilde{S}}_{2}$ and ${\tilde{S}}_{3}$ are given by using (A40) and (A41).

Considering the natures of ${\bar{A}}_{j}$ in (A5), ${\bar{C}}_{j}$ in (A3), and ${\tilde{C}}_{j}$ in (A42), we can see from a statement by [34] (Lemma 2) that if we choose the variables $x_{k}$ as $H^{\left[ji\right]}\left(\omega_{t}\right),H_{T-\mathrm{IR}}^{\left[ri^{\prime }\right]}\left(\omega_{t}\right),i,i^{\prime },j\in \left\{1,\ldots ,K\right\},u\in \left\{1,\ldots ,Q\right\}$, $y_{k}$ as $H_{\mathrm{IR}-R}^{\left[ju\right]}\left(\omega_{t}\right),j\in \left\{U+1,\ldots ,K\right\},u\in \left\{1,\ldots ,Q\right\}$, and $z_{k}$ as $H_{\mathrm{IR}-R}^{\left[ju\right]}\left(\omega_{t}\right),j\in \left\{1,\ldots ,U\right\},u\in \left\{1,\ldots ,Q\right\}$, then by using [34] (Lemmas 1–3), subspaces ${\bar{A}}_{j}$, ${\bar{C}}_{j}$ and ${\tilde{C}}_{j}$ are full-rank and linearly independent (all base vectors of these subspaces are linearly independent) almost surely. The reason is that if we take the constructing base vectors of ${\bar{A}}_{j}$, ${\bar{C}}_{j}$, and ${\tilde{C}}_{j}$ and construct a square matrix by choosing some rows of it, we can see by using [34] (Lemmas 2–3) that the determinant of this square matrix is a non-zero polynomial, which is non-zero with the probability 1 by using [34] (Lemma 1). Thus, all the message streams are decodable at the clean receivers (by using zero forcing). For more clarity, [34] (Lemmas 1–3) are reviewed in Appendix B.

Similar to the proof of Theorem 1, first we assume that the parameter *T* is sufficiently large, and at the end of this step, we determine the minimum required *T*. The dimensions of the subspaces ${\bar{C}}_{j}$ and ${\bar{A}}_{j}$ are the same as (A8) and (A10), respectively. Hence, we calculate the dimension of ${\tilde{C}}_{j}$ by calculating the number of its base vectors in (A42) as follows:

(A43) $$d\left({\tilde{C}}_{j}\right)=n^{K^{2}-K}{\left(sn\right)}^{\varphi }{\left(\upsilon n\right)}^{\theta },$$

where

$$\varphi =\sum_{q^{\prime }=1}^{Q}\left(K-\left|E_{L(q^{\prime })}\right|\right)=KQ-\sum_{q^{\prime }=1}^{Q}\left|E_{L(q^{\prime })}\right|=KQ-U^{2},$$

$$\theta =\sum_{q^{\prime }=1}^{Q}\left|E_{L(q^{\prime })}\right|=U^{2}.$$

We can see from (10) that $l=K^{2}-K+QK$. We can define the following parameters:

$$\Gamma =s^{QK},$$

$$\chi =s^{QK-U^{2}}\upsilon^{U^{2}},$$

$$\zeta =t^{QK}.$$

Therefore, the normalized asymptotic dimensions of the message and interference subspaces are:

(A44) $$D_{N}\left({\bar{C}}_{j}\right)=\Gamma ,$$

(A45) $$D_{N}\left({\tilde{C}}_{j}\right)=\chi ,$$

(A46) $$D_{N}\left({\bar{A}}_{j}\right)=\max\left\{\Gamma ,\zeta \right\}.$$

Thus, interference alignment Equations (24) and (25) are satisfied.

*(2) Validity of interference alignment conditions at the dirty receivers j∈{U+1,…,K}:* For the dirty receivers, the message subspace ${\bar{C}}_{j}$ and the interference subspace ${\bar{A}}_{j}$ are exactly the same as (A17) and (A18). To satisfy interference alignment Equation (28) (if *W* is replaced with *U*), the subspace ${\tilde{A}}_{j}$ must be chosen such that:

$$\underset{i\in \{1,\ldots ,U\}}{⋃}\left\{\mathrm{span}\left({\tilde{H}}^{\left[ji\right]}{\tilde{V}}^{\left[i\right]}\right)\right\}\subseteq {\tilde{A}}_{j}.$$

Therefore, we can characterize subspace ${\tilde{A}}_{j}$ as follows:

$${\tilde{A}}_{j}=\mathrm{span}\left\{\left[M(g_{1}\left(i,j\right),{\tilde{H}}^{\left[ji\right]},{\bar{S}}_{1})\right]\left[M(g_{2}\left(i,q\right),H_{T-\mathrm{IR}}^{\left[qi\right]},{\tilde{S}}_{2})\right]\left[M(g_{3}\left(i,q\right),T^{\left[qi\right]},{\tilde{S}}_{3})\right]w:\right.$$

(A47) $$g_{1}\in F\left({\bar{S}}_{1},\left\{1,\ldots ,n+1\right\}\right),g_{2}\in F\left({\tilde{S}}_{2},\left\{1,\ldots ,sn\right\}\right)\left.,g_{3}\in F\left({\tilde{S}}_{3},\left\{1,\ldots ,\upsilon n\right\}\right)\right\},$$

where ${\tilde{S}}_{2}$ and ${\tilde{S}}_{3}$ are given by using (A40) and (A41).

By using the same argument given for ${\bar{A}}_{j}$, ${\bar{C}}_{j}$ and ${\tilde{C}}_{j}$ at the clean receivers, subspaces ${\bar{A}}_{j}$, ${\tilde{A}}_{j}$ and ${\bar{C}}_{j}$ are full-rank and linearly independent almost surely. Then, we have:

(A48) $$D_{N}\left({\bar{C}}_{j}\right)=\zeta ,$$

(A49) $$D_{N}\left({\bar{A}}_{j}\right)=\max\left\{\Gamma ,\zeta \right\},$$

(A50) $$d\left({\tilde{A}}_{j}\right)={\left(n+1\right)}^{K^{2}-K}{\left(sn\right)}^{QK-U^{2}}{\left(\upsilon n\right)}^{U^{2}},$$

(A51) $$D_{N}\left({\tilde{A}}_{j}\right)=\chi .$$

Hence, we can see that interference alignment Equations (26)–(29) are satisfied.

*(3) Validity of interference alignment conditions at the q-th antenna of the NC-IR q∈{1,…,Q}*:The interference subspace ${\bar{A}}_{r_{q}}$ is exactly the same as (A24) if we replace *W* with *U*. The message subspaces ${\tilde{C}}_{i,r_{q}},i\in E_{L(q)}$ and the interference subspace ${\tilde{A}}_{r_{q}}$ will change as follows:

$${\tilde{C}}_{i,r_{q}}=\mathrm{span}\left(H_{T-\mathrm{IR}}^{\left[qi\right]}{\tilde{V}}^{\left[i\right]}\right)=$$

$$\mathrm{span}\left\{H_{T-\mathrm{IR}}^{\left[qi\right]}\left[M(g_{1}\left(i,j\right),{\tilde{H}}^{\left[ji\right]},{\bar{S}}_{1})\right]\left[M(g_{2}\left(i,q\right),H_{T-\mathrm{IR}}^{\left[qi\right]},{\tilde{S}}_{2})\right]\left[M(g_{3}\left(i,q\right),T^{\left[qi\right]},{\tilde{S}}_{3})\right]w:\right.$$

(A52) $$g_{1}\in F\left({\bar{S}}_{1},\left\{1,\ldots ,n\right\}\right),g_{2}\in F\left({\tilde{S}}_{2},\left\{1,\ldots ,sn\right\}\right)\left.,g_{3}\in F\left({\tilde{S}}_{3},\left\{1,\ldots ,\upsilon n\right\}\right)\right\},$$

where ${\tilde{S}}_{2}$ and ${\tilde{S}}_{3}$ are given by using (40) and (41), respectively.

$${\tilde{C}}_{i,r_{q}}=\mathrm{span}\left(H_{T-\mathrm{IR}}^{\left[qi\right]}{\tilde{V}}^{\left[i\right]}\right)=$$

$$\mathrm{span}\left\{H_{T-\mathrm{IR}}^{\left[qi\right]}\left[M(g_{1}\left(i,j\right),{\tilde{H}}^{\left[ji\right]},{\bar{S}}_{1})\right]\left[M(g_{2}\left(i,q\right),H_{T-\mathrm{IR}}^{\left[qi\right]},{\tilde{S}}_{2})\right]\left[M(g_{3}\left(i,q\right),T^{\left[qi\right]},{\tilde{S}}_{3})\right]w:\right.$$

(A53) $$g_{1}\in F\left({\bar{S}}_{1},\left\{1,\ldots ,n\right\}\right),g_{2}\in F\left({\tilde{S}}_{2},\left\{1,\ldots ,sn\right\}\right)\left.,g_{3}\in F\left({\tilde{S}}_{3},\left\{1,\ldots ,\upsilon n\right\}\right)\right\},$$

where ${\tilde{S}}_{2}$ and ${\tilde{S}}_{3}$ are given by using (A40) and (A41), respectively.

To satisfy interference alignment Equation (A35), the subspace ${\tilde{A}}_{r_{q}}$ must be chosen such that:

$$\underset{i\in \left\{1,\ldots ,U\right\},i\notin E_{L(q)}}{⋃}\left\{\mathrm{span}\left(H_{T-\mathrm{IR}}^{\left[qi\right]}{\tilde{V}}^{\left[i\right]}\right)\right\}\subseteq {\tilde{A}}_{r_{q}}.$$

Therefore, we can characterize ${\tilde{A}}_{j}$ as follows:

$${\tilde{A}}_{r_{q}}=\mathrm{span}\left\{\left[M(g_{1}\left(i,j\right),{\tilde{H}}^{\left[ji\right]},{\bar{S}}_{1})\right]\left[M(g_{2}\left(i,q\right),H_{T-\mathrm{IR}}^{\left[qi\right]},{\tilde{S}}_{2})\right]\left[M(g_{3}\left(i,q\right),T^{\left[qi\right]},{\tilde{S}}_{3})\right]w:\right.$$

(A54) $$g_{1}\in F\left({\bar{S}}_{1},\left\{1,\ldots ,n\right\}\right),g_{2}\in F\left({\tilde{S}}_{2},\left\{1,\ldots ,sn+1\right\}\right)\left.,g_{3}\in F\left({\tilde{S}}_{3},\left\{1,\ldots ,\upsilon n\right\}\right)\right\},$$

where ${\tilde{S}}_{2}$ and ${\tilde{S}}_{3}$ are given by using (A40) and (A41), respectively.

By using the same argument given before, subspaces ${\bar{A}}_{r_{q}}$, ${\tilde{A}}_{r_{q}}$, and ${\tilde{C}}_{i,r_{q}},i\in E_{L(q)}$ are full-rank and linearly independent almost surely. We can see that:

(A55) $$d\left({\tilde{C}}_{i,r_{q}}\right)=n^{K^{2}-K}{\left(sn\right)}^{QK-U^{2}}{\left(\upsilon n\right)}^{U^{2}},\forall i\in E_{L(q)},$$

(A56) $$D_{N}\left({\tilde{C}}_{i,r_{q}}\right)=\chi ,$$

so the normalized dimension of the total subspaces that can be de-multiplexed at the NC-IR *q*-th antenna is:

(A57) $$\sum_{i\in E_{L(q)}}D_{N}\left({\tilde{C}}_{i,r_{q}}\right)=\left|E_{L(q)}\right|\chi .$$

For ${\bar{A}}_{r_{q}}$, the same as in the proof of Theorem 1, we have:

(A58) $$D_{N}\left({\bar{A}}_{r_{q}}\right)=\max\left\{\Gamma ,\zeta \right\}.$$

For ${\tilde{A}}_{r_{q}}$, we have:

(A59) $$d\left({\tilde{A}}_{r_{q}}\right)=n^{K^{2}-K}{\left(sn+1\right)}^{QK-U^{2}}{\left(\upsilon n\right)}^{U^{2}},$$

(A60) $$D_{N}\left({\tilde{A}}_{r_{q}}\right)=\chi .$$

Thus, we can see that interference alignment Equations (30)–(33) are satisfied.

The same as in the proof of scheme 1 in Theorem 1, we derive the dimension of the whole received signal space at each receiver. Therefore, if we define $d_{t,j}$ as the total dimension at the *j*-th receiver and $d_{t,r_{q}}$ as the total dimension at the *q*-th receiving antenna of the NC-IR, then we can see (53)–(55) will be obtained if we replace *W* and $B_{q}$ with *U* and $E_{L(q)}$, respectively. Therefore, considering $D_{N,t,j}$ as the total normalized asymptotic dimension at the *j*-th receiver and $D_{N,t,r_{q}}$ as the total normalized asymptotic dimension at the *q*-th antenna of the NC-IR, we have:

(A61) $$D_{N,t,j}=\Gamma +\chi +\max\left\{\Gamma ,\zeta \right\},\forall j\in \left\{1,\ldots ,U\right\},$$

(A62) $$D_{N,t,j}=\zeta +\chi +\max\left\{\Gamma ,\zeta \right\},\forall j\in \left\{U+1,\ldots ,K\right\},$$

(A63) $$D_{N,t,r_{q}}=\left|E_{L(q)}\right|\chi +\chi +\max\left\{\Gamma ,\zeta \right\},\forall q\in \left\{1,\ldots ,Q\right\}.$$

Considering the parameter *T* as (59), we have:

(A64) $$\lim_{n\to \infty }\frac{T}{n^{K^{2}-K+QK}}=\chi +\max\left\{\Gamma ,\zeta \right\}+\max\left\{\max_{q\in \{1,\ldots ,Q\}}\left|E_{L(q)}\right|\chi ,\zeta ,\Gamma \right\}.$$

Moreover, we have:

(A65) $$\max_{q\in \{1,\ldots ,Q\}}\left|E_{L(q)}\right|=\left\lceil \frac{U}{p}\right\rceil .$$

Therefore, from using (A64) and (A65), we can conclude that:

(A66) $$\lim_{n\to \infty }\frac{T}{n^{K^{2}-K+QK}}=\chi +\max\left\{\Gamma ,\zeta \right\}+\max\left\{\left\lceil \frac{U}{p}\right\rceil \chi ,\zeta ,\Gamma \right\}.$$

Moreover we let:

(A67) Γ=ζ,

(A68) $$\zeta \geq \left\lceil \frac{U}{p}\right\rceil \chi .$$

By using assumptions (A67) and (A68), we can see that the total normalized length is:

(A69) $$\lim_{n\to \infty }\frac{T}{n^{K^{2}-K+QK}}=\chi +2\Gamma .$$

**Step 6: DoF Analysis**

Now, we can characterize the total DoF. As stated before, we have *U* clean receivers, each with a normalized message dimension equal to Γ+χ, and K−U dirty receivers, each with a normalized message dimension equal to ζ (note that we assumed ζ=Γ). Therefore, the total normalized length of *T* is equal to χ+2Γ. Thus, the total DoF has the following form:

(A70) $$\mathrm{DoF}=\max_{\chi \geq 0,\Gamma \geq \left\lceil \frac{U}{p}\right\rceil \chi }\frac{U(\chi +\Gamma )+(K-U)\Gamma }{\chi +2\Gamma }.$$

By assuming that Γ=βχ, we have:

(A71) $$\begin{array}{cc} \mathrm{DoF} & =\max_{\beta \geq \left\lceil \frac{U}{p}\right\rceil }\frac{U(1+\beta )+(K-U)\beta }{1+2\beta } \end{array}$$

(A72) $$\begin{array}{cc} \; & =\frac{K}{2}+\max_{\beta \geq \left\lceil \frac{U}{p}\right\rceil }K\frac{\frac{U}{K}-\frac{1}{2}}{1+2\beta }=\frac{K}{2}+\max\left\{K\frac{\frac{U}{K}-\frac{1}{2}}{1+2\left\lceil \frac{U}{p}\right\rceil },0\right\}, \end{array}$$

where (A72) follows from the fact that if $\frac{U}{K}>\frac{1}{2}$, we set $\beta =\left\lceil \frac{U}{p}\right\rceil$, and if $\frac{U}{K}<\frac{1}{2}$, we tend β to *∞*. This completes the proof.
